# Metabolic Profiling of Sugarloaf Chicory Roots: Structural Assignment of Sesquiterpene Lactone Conjugates and Response to Reduced Irrigation

**DOI:** 10.3390/molecules31040712

**Published:** 2026-02-19

**Authors:** Giuseppe Scioli, Lorenzo Pin, Giulio Testone, Anatoly Petrovich Sobolev, Donato Giannino

**Affiliations:** 1 Institute for Biological Systems, Italian National Research Council, Monterotondo, 00015 Rome, Italy; giuseppescioli@cnr.it (G.S.); lorenzo.pin@cnr.it (L.P.); giulio.testone@cnr.it (G.T.); 2 Institute for Agricultural and Forestry Systems in the Mediterranean, Catania Unit, 95128 Catania, Italy

**Keywords:** Sugarloaf chicory (*Cichorium intybus* var. *porphyreum*), quantitative ^1^H NMR metabolite profiling, sesquiterpene lactones, root water deficit

## Abstract

Sugarloaf chicory (*Cichorium intybus* var. *porphyreum*) represents a valuable crop for investigating metabolic responses to environmental stress. This study applied quantitative ^1^H-NMR-based metabolomics to characterize the water-soluble metabolome and evaluate root metabolic adaptations under water-deficit (WD) conditions compared to well-watered (WW) conditions. A total of 44 compounds were identified across roots and leaves, with inulin being root-specific. To address the lack of aqueous NMR data for chicory sesquiterpene lactones (STLs), a solid-phase extraction and fractionation protocol was implemented. Comparison of ^1^H-NMR and ^13^C chemical shifts with data from the literature, 2D NMR experiments (HSQC, HMBC), and spiking with standards confirmed that the major root STLs (lactucin, 8-deoxylactucin, and lactucopicrin) are 15-oxalate conjugates with enhanced water solubility. Under water deficit, root profiles revealed significant stress-induced alterations: sucrose, alanine, threonine and phospho-choline increased, whereas asparagine, glutamic acid, chiro-inositol, myo-inositol, and all three STL conjugates decreased markedly (−39% to −50%). These shifts reflect adaptive osmotic adjustments and carbon reallocation strategies under stress. As roots represent a remarkable source of bioactive STLs, these findings support their potential valorization as functional ingredients. This study establishes quantitative NMR metabolomics as a robust tool for assessing physiological responses to water deficit, providing insights into stress adaptation mechanisms and identifying roots as promising targets for alternative applications.

## 1. Introduction

Cultivated forms of *Cichorium intybus* were originally classified [1] into var. *foliosum* (leafy/salad use) and var. *sativum* (industrial roots), recently superseded by genetic/horticultural criteria distinguishing specific biotypes for aerial parts [2]: Radicchio (var. *latifolium*), Witloof/Belgian endive (var. *foliosum*), Catalogna (var. *sylvestre*), and Sugarloaf (var. *porphyreum*). The latter (synonyms “Pan de sucre”, “Pan di Zucchero”), originating from Northern Italy [3], is currently widely cultivated across Central Europe, where it serves as a crucial seasonal crop for the winter fresh and processing markets. Metabolically, it is distinctive among leaf chicories for having the highest total sesquiterpene lactone content and the lowest total phenolic content in leaves; notably, it lacks anthocyanins, with hydroxycinnamic acids comprising the majority of its phenolic pool and flavonoids composed primarily of kaempferol and quercetin [4].

Further research is necessary to widen aspects either on leaf quality and on taproots, which are generally discarded, despite representing a potentially valuable side stream rich in inulin and bioactive compounds for circular food chains [5,6]. Root chicory (*C. intybus* var. *sativum*) is industrially exploited as a major source of inulin, a prebiotic fructan that constitutes 44–68% of root dry weight depending on cultivar and growing conditions, with peaks exceeding 70% [7]. Due to its prebiotic properties and functional characteristics, inulin is widely used as a low-calorie bulking agent and fat replacer [8]. Roots of chicories can accumulate bitter, guaianolide-type sesquiterpene lactones (STLs), mainly 8-deoxylactucin, lactucin and lactucopicrin as oxalate conjugates (8-DEOLCT-OX15, LCT-OX15, LCP-OX15). STLs contribute to plant defense, regulate bitterness in edible parts, and have antimicrobial, anti-inflammatory, and anthelmintic activities exploitable in medicine [9,10,11]. If water stress is known to modulate inulin content, its degree of polymerization and carbohydrate partitioning in Witloof root [12], analogous information for leaf-type chicories, and specifically for Sugarloaf, is scarce, and far less is known about coordinated adjustments in amino-acid, organic-acid and STL metabolism in roots of fresh-market cultivars grown under realistic field irrigation scenarios.

^1^H-NMR-based untargeted metabolomics offers a holistic fingerprint of primary and secondary metabolites, providing highly reproducible quantitative data and simultaneous structural assignment without compound-specific calibration curves, an approach increasingly applied to relate crop profiles to genotype and environment [13]. While NMR profiling has characterized diverse chicory materials, from roasted roots [14] to saline-stressed spiny chicory [15] and local leaf varieties [16]), most data on chicory sesquiterpene lactones (STLs) still rely on HPLC or LC–MS methods [4,17]. NMR has emerged as a powerful tool for both structural elucidation and direct quantification of STLs in complex extracts [10,11].

Unlike prior LC–MS-based STL studies, in this work, ^1^H-NMR untargeted metabolomics was applied for direct, calibration-free quantification of sesquiterpene lactones alongside primary metabolites in Sugarloaf organs. Here, 44 water-soluble compounds were assigned, filling gaps in STLs oxalate quantification and inulin (including fructose units). Additional objectives included assessing differences in metabolite composition (narrowed to 38 compounds) between leaves and roots and evaluating the impact of reduced irrigation on metabolite abundance in roots. Overall, water deficit induced coordinated alterations in stress-responsive amino acids, carbohydrates, inositols and STL depletion. These outcomes provided insight into water stress mechanisms and offer implications for crop management and root biomass valorization.

## 2. Results

### 2.1. Spectral Assignment of Hydro Soluble Fraction

NMR spectra were assigned and water-soluble metabolites identified following a previously established procedure [18] for stems of *C. intybus* var. *sylvestre* (biotype “Catalogna”), based on 2D NMR experiments (TOCSY, HSQC, HMBC) on leaf (Supplementary Figures S1–S3) and root extracts (Supplementary Figures S4–S6) and comparison with NMR data for common plant metabolites and related chicory varieties. The strength of all assignments and the specific metabolite identification relied on the complete pattern of C-H correlations for each molecule obtained in HSQC experiments (Supplementary Figures S2 and S5), matching data from the literature and the database of single-compound spectra. This means that random coincidences of single signals from different compounds cannot lead to the wrong assignments.

A total of 44 metabolites were identified, with 35 shared by leaves and roots. Glycine, pyroglutamic acid, galactose, raffinose, fructose, chicoric acid, monocaffeoyltartaric acid, and trigonelline occurred only in leaves, whereas inulin occurred only in roots.

**Figure 1:**
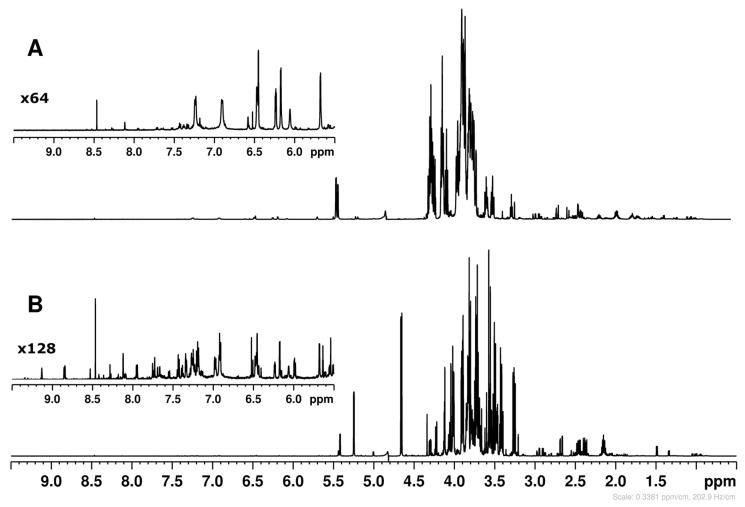
^1^H NMR spectra of Sugarloaf root (**A**) and leaf (**B**) extracts. (**A**) Entire proton spectrum and expansion of aromatic region of root extract. (**B**) Entire proton spectrum and expansion of aromatic region of leaf extract.

Typical ^1^H NMR spectra of Sugarloaf leaf and root extracts are shown in Figure 1. The leaf spectrum is dominated by sugar signals in the 5.5–3.0 ppm region, whereas the root spectrum exhibits very intense inulin oligomer signals in the same region. As for carbohydrates, anomeric proton signals in the 5.5–4.5 ppm region indicate raffinose (5.43 and 5.00 ppm), sucrose (5.42 ppm), α- and β-glucose (5.24 and 4.66 ppm), and α- and β-galactose (5.27 and 4.59 ppm); three abundant fructose anomeric forms were also identified (Table 1). Inulin, detected only in the root extract, showed the characteristic anomeric proton signal of the terminal α-glucose at 5.44 ppm and consists of oligofructans with terminal glucosyl and β-(2,1)-linked fructosyl units. Its degree of polymerization (DP), which affects solubility, sweetness and other properties, was estimated by two methods: diffusion-ordered spectroscopy (DOSY, Supplementary Figure S7), using the self-diffusion coefficient of inulin signals, and the fructose-to-glucose ratio calculated from the integrals of the glucose anomeric proton at 5.44 ppm and fructose H-3 protons at 4.21–4.28 ppm, yielding similar mean DP values. DOSY showed that the average number of fructose units in the chain was 7.9, compared to 7.5 for the same sample, calculated as the fructose-to-glucose ratio.

Among other metabolites, 17 amino acids, eight organic acids, three amino alcohols and three isomeric polyols were identified (Table 1). Chicoric and monocaffeoyltartaric acids, polyphenols typical for *Cichorium* spp., were also identified in leaf extracts, whereas other frequently occurring polyphenols like chlorogenic acid were not detected.

### 2.2. Assignment and Identification of Sesquiterpene Lactone Oxalates

The ^1^H NMR spectra showed unknown resonances between 7.5 and 5.5 ppm, which were most intense in root extracts (Figure 1A). This region typically contains CH protons of aromatic rings and double bonds; in *C. intybus*, it includes aromatic amino acids, polyphenols and, notably, sesquiterpene lactones (STLs), which are abundant in roots but poorly soluble in water unless conjugated with glucose or oxalic acid [19]. Because NMR data for STLs in aqueous solution are not available, an additional identification step was implemented: the STL fraction was retained on an SPE cartridge, eluted in three fractions of increasing hydrophobicity using mixed water/methanol solvents with varying ratios of methanol (from 20% to 100%), and each fraction was dissolved in deuterated methanol to assign signals and identify compounds by comparison with published NMR data [20].

#### 2.2.1. Assignment of STLs in Methanol Solution

**Figure 2:**
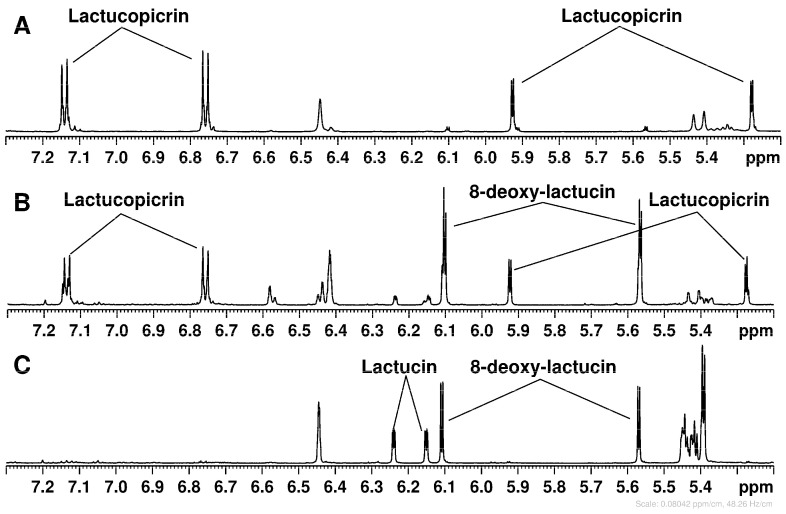
^1^H NMR spectra of three SPE fractions of the Sugarloaf root extract dissolved in CD_3_OD. (**A**) 100% MeOH fraction, (**B**) 1:1 (*v*/*v*) H_2_O:MeOH fraction, and (C) 8:2 (*v*/*v*) H_2_O:MeOH fraction.

**Figure 3:**
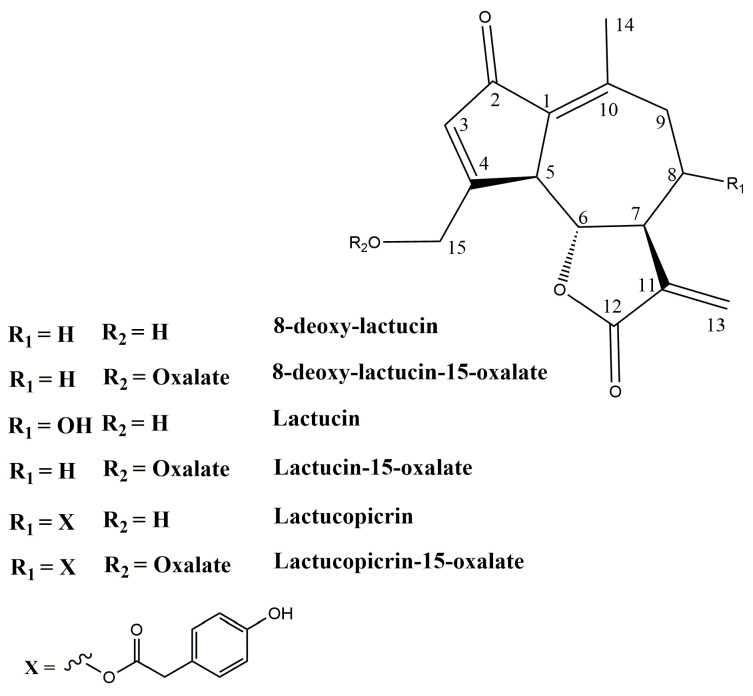
Chemical structure of sesquiterpene lactones.

Figure 2 shows the ^1^H NMR spectra of three SPE fractions dissolved in deuterated methanol. The region 7.5–5.2 ppm shown with the vertical expansion in Figure 2A–C, exhibits characteristic signals of terminal double bonds =CH_2_ present in lactucin, lactucopicrin, and 8-deoxylactucin [20]; see also Figure 3. Taking these signals as a point of departure, the assignment of complete sets of ^1^H and ^13^C NMR signals was performed by means of the correlation data from 2D NMR experiments (^1^H-^1^H TOCSY, ^1^H-^13^C HSQC, and ^1^H-^13^C HMBC) carried out for each fraction (Table 2). The comparison of the spectra in Figure 2 clearly indicates that the separation of STL between the various fractions was only partial, with each STL distributed across two fractions. Specifically, lactucopicrin was detected in the 1:1 (*v*/*v*) H_2_O:MeOH and 100% MeOH fractions, while lactucin and 8-deoxylactucin were present in both the 8:2 (*v*/*v*) H_2_O:MeOH and 1:1 (*v*/*v*) H_2_O:MeOH fractions. Anyway, even a partial separation of STL was useful for the simplification of the assignment process, especially in the case of the 100% MeOH fraction, wherein only lactucopicrin was detected.

A methodical comparison of the ^1^H and ^13^C chemical shifts in three STLs identified in SPE fractions was approached using assignments from chicory for the non-conjugated forms of lactucin, lactucopicrin, and 8-deoxy-lactucin [20]. Inconsistencies were observed both in the assignment within each standard (Table 2, notes b-e) and between the STLs identified in our analysis and those reported in the literature. Notably, six ^13^C signals were interchanged for lactucin and 8-deoxylactucin in methanol solution [20], and the erroneous assignment of the ^13^C chemical shifts for C-4 in lactucopicrin-15-oxalate and lactucin-15-oxalate was also reported [21]. The ^1^H chemical shifts in both diastereotopic protons of the CH_2_-15 group were found to be 0.7–0.5 ppm higher than the literature values (Table 2). A less pronounced yet still significant increase (0.17–0.14 ppm) in the corresponding ^1^H chemical shift in the CH-5 group was also observed, as well as a less pronounced yet still significant increase (1.7–1.2 ppm) in the corresponding ^13^C chemical shift for the same group. Consequently, these differences were ascribed to the conjugation of STLs to oxalate. The downfield shifts of ^1^H and ^13^C NMR signals of the CH_2_OH-15 group esterified with oxalic acid indicate the deshielding of corresponding nuclei as a consequence of the electronegative effect of the oxalate. The NMR data on lactucopicrin-15-oxalate and lactucin-15-oxalate dissolved in DMSO [19,21] confirmed the observed downfield shifts of ^1^H and ^13^C NMR signals of the CH_2_OH-15 group. Unfortunately, the instability of the methanol solution and relatively low concentration of STLs made direct observation of ^13^C NMR data impossible. However, the long-range correlation between CH_2_-15 protons and the carbon of the esterified carboxyl group of oxalate was observed in HMBC spectra for 8-deoxylactucin-15-oxalate and lactucin-15-oxalate. Conjugation with oxalate explains why lactucopicrin is soluble in aqueous solution, when it would otherwise be insoluble.

Another consequence of esterification with oxalate is the “gamma effect”, where steric repulsion between a substituent and the γ-carbon creates an upfield shift in the ^13^C NMR signal observed for C-4 carbon. In fact, for all three STLs, the C-4 ^13^C signals were detected at 168–169 ppm upon conjugation, rather than at 176 ppm, as observed in non-conjugated molecules.

#### 2.2.2. Assignment of STL NMR Spectra in Buffered D_2_O Solution

**Figure 4:**
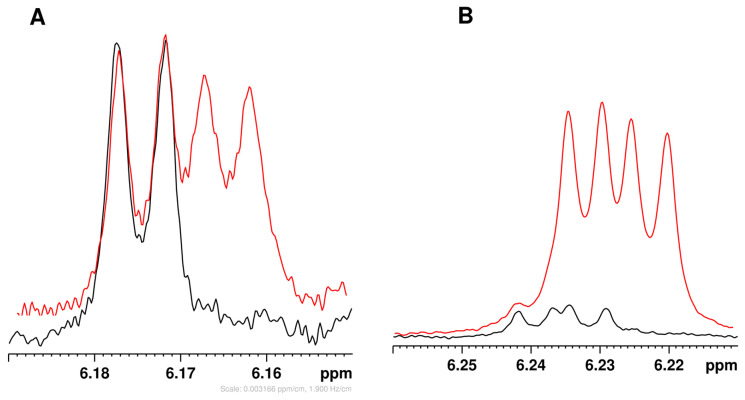
Representative spectral regions showing spiking experiments for the CH_2_-13 resonance of (**A**) 8-deoxy-lactucin and (**B**) lactucin. The original extracts are in black, while the spectra following standard addition are in red.

The same SPE fractions containing sesquiterpene lactones were dissolved in buffered D_2_O (pH 7.0) and analyzed using 2D NMR (TOCSY, HSQC, and HMBC) for ^1^H and ^13^C signal assignment. These assignments are reported in Table 3, where they are compared with the NMR data of non-conjugated STL dissolved in the same buffered D_2_O solution. Lactucopicrin’s poor solubility limited analysis to proton spectra. The observed NMR signals confirmed the presence of lactucin-, 8-deoxylactucin-, and lactucopicrin-15-oxalate conjugates, indicated by downfield shifts in CH_2_OH-15 protons (0.60–0.70 ppm) and carbons (2–3 ppm), with HMBC verifying long-range correlations between CH_2_-15 protons and the carbon of the esterified carboxyl group of oxalates (at 165.0 ppm). These conjugates were also detected in whole root and leaf extracts. Spiking root extracts with lactucin and 8-deoxylactucin standards confirmed lactucin-15-oxalate and 8-deoxylactucin-15-oxalate presence (Figure 4); lactucopicrin spiking was ineffective due to low solubility.

#### 2.2.3. STL Hydrolysis/Transformation

Conjugated STLs were unstable in both water and methanol. In methanol, oxalate hydrolysis produced non-conjugated STLs. In buffered D_2_O, the loss of conjugated STLs was not matched by a proportional rise in non-conjugated forms, indicating that hydrolysis explains the behavior only partly. Additionally, 4-hydroxyphenylacetic acid, a degradation product of lactucopicrin-15-oxalate, was detected in aqueous solutions.

### 2.3. Metabolite Content Differences in Leaves and Roots

Quantification of 38 metabolites (out of 44 identified in the spectra) was completed for leaves and roots (Table 4). Glycine, pyroglutamic acid, lysine, and succinic acid lacked non-overlapping ^1^H signals, preventing reliable measurement. Signals from chicoric and monocaffeoyltartaric acids were too weak for accurate quantification due to noise. Significant content differences between organs were assessed by one-way ANOVA.

#### 2.3.1. Amino Acids and Amino Alcohols

Among amino acids (AA), 14 were quantified in leaves and 13 in roots. Glutamine and arginine were the most abundant residues in leaves and roots, respectively (both >13 mg g^−1^ DW). Five of the 13 shared AA did not differ significantly between tissues. Alanine, asparagine, aspartic acid, gamma-aminobutyric acid, glutamine, and glutamic acid were higher in leaves, whereas arginine, leucine, and valine were higher in roots. Notably, arginine levels were ~40-fold higher in roots than in leaves, while alanine and glutamic acid were more than 3-fold higher in leaves than in roots.

The content rank order of choline, followed by ethanolamine and phosphocholine, was maintained in both tissues. However, phosphocholine levels were more than 3-fold higher in leaves than in roots.

#### 2.3.2. Organic Acids

The tricarboxylic (citric, fumaric, malic, and succinic) and other organic (lactic, formic, tartaric, and quinic) acids showed significantly different contents between organs. In leaves, citric, tartaric, malic, and quinic acids were most abundant (in descending order), whereas in roots, the most abundant were citric, malic, and quinic acids. Most of these were higher in leaves than roots, except for citric acid, which showed the opposite trend.

#### 2.3.3. Carbohydrate and Sugar Alcohol Content

In leaves, glucose and fructose were the most abundant sugars, followed by sucrose, raffinose, and β-galactose, while inulin was not detected. Inulin was root-specific and represented the major carbohydrate, followed by sucrose and glucose; the remaining sugars were undetected. The absence of detectable fructose in roots is explained by its extensive storage/conversion into inulin. Inulin accounted for more than 50% of the dry weight in roots, where sucrose was 10-fold lower than inulin but twice as abundant as in leaves. Among the three isomeric sugar alcohols, myo-inositol predominated in leaves (over 2.5-fold higher than chiro- and scyllo-inositol), whereas in roots, chiro- and myo-inositol were nearly equal and 5-fold higher than scyllo-inositol. Chiro-inositol was markedly enriched in roots and likely characterizes this tissue.

#### 2.3.4. Sesquiterpene Lactone Content

The content was 8- to 20-fold higher in roots than in leaves, where the three compounds were equally distributed, while in roots, 8-deoxy-lactucin-15-oxalate was the major constituent.

### 2.4. Content Variation in Roots in Reduced Irrigation Regime

**Figure 5:**
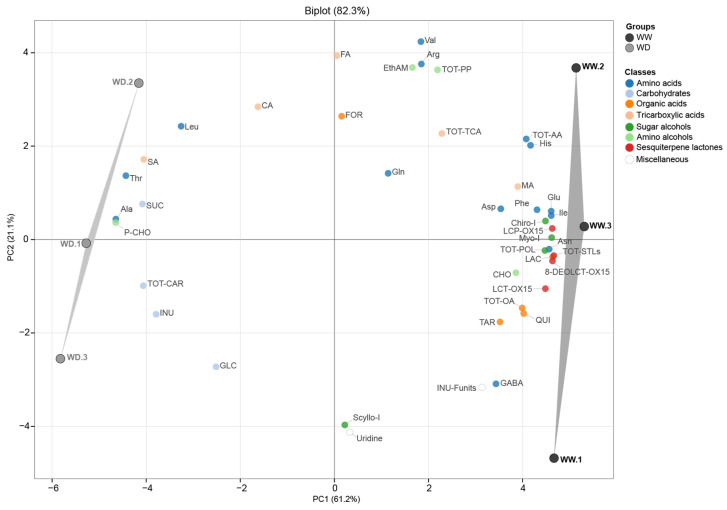
PCA biplot displaying the distribution of 42 metabolites (34 individual compounds and 8 class totals) in Sugarloaf roots harvested from plants grown under well-watered (WW, full irrigation) and water-deficit (WD, reduced irrigation of 67%) conditions. Legend: Leu, leucine; Ile, isoleucine; Val, valine; Thr, threonine; Ala, alanine; Arg, arginine; Glu, glutamic acid; Gln, glutamine; Asp, aspartic acid; Asn, asparagine; Phe, phenylalanine; His, histidine; GABA, gamma-aminobutyric acid; TOT−AA, total amino acids; CA, citric acid; MA, malic acid; FA, fumaric acid; SA, succinic acid; TOT-TCA, total tricarboxylic acids; GLC, glucose; SUC, sucrose; INUL, inulin; INU−F, number of fructose units in inulin; TOT−CAR, total carbohydrates; EthAM, ethanolamine; CHO, choline; P−CHO, phosphocholine; TOT−PP, total precursors of phospholipids; Myo.I, myo-inositol; Scyllo.I, scyllo−inositol; Chiro.I, chiro−inositol; TOT-POL, total polyols; FOR, formic acid; TAR, tartaric acid; LA, lactic acid; QUI, quinic acid; TOT−OA, total organic acids; 8-DEOLCT-OX15, 8−deoxylactucin; LCT−OX15, lactucin; LCP−OX15, lactucopicrin; TOT−STLs, total sesquiterpene lactones; URI, uridine.

Following metabolite assignment and quantification, an untargeted approach assessed content variations in 34 water-soluble root metabolites, plus inulin degree of polymerization (DP), in response to water deficit. Principal Component Analysis (PCA, Figure 5) revealed that PC1 and PC2 explained 61.22% and 21.17% of the total variance, respectively. The biplot showed clear separation between treatments, with samples from well-watered (WW) and water-deficit (WD) roots clustering in the positive and negative quadrants of PC1, respectively. WD samples (left group) were characterized by high levels of sucrose, alanine, threonine, and succinic acid, while WW samples (right group) by high levels of sesquiterpene lactones, asparagine, and glutamic acid.

ANOVA results (Table 5) indicated that water deficit significantly affected the levels of 16 metabolites. Specifically, amino acid profiles shifted, with significant increases in Ala (+79%) and Thr (+17%) and decreases in Ile (−35%), Asn (−29%), His (−20%), and Glu (−16%). Among TCA intermediates and sugars, succinic acid and sucrose increased by over 19%, while quinic acid derivative and chiro- and myo-inositol levels declined by over 20%. Notably, all three sesquiterpene lactones decreased markedly (−40 to −51%) under water reduction.

### 2.5. Metabolite Relationships

**Figure 6:**
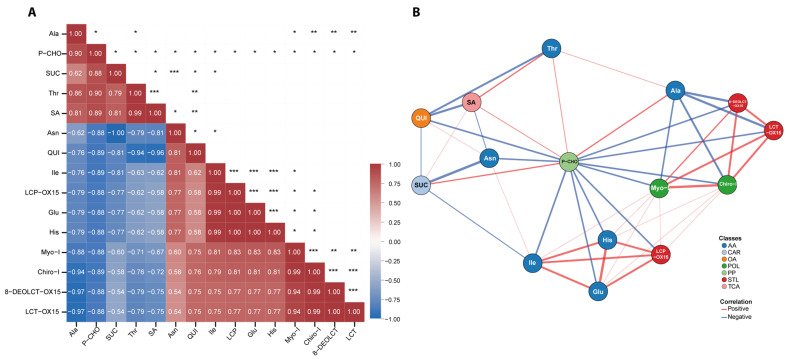
Correlation analysis of metabolite profiles. (**A**) Correlogram visualizing metabolite–metabolite relationships. Squares display the Spearman correlation coefficient, colored according to intensity from negative (blue) to positive (red). Asterisks denote statistical significance (*, *p* ≤ 0.05; **, *p* ≤ 0.01; ***, *p* ≤ 0.001). (**B**) Correlation network was constructed using threshold values of r ≥ |0.7| and *p* ≤ 0.01. Line width indicates correlation strength; blue and red edges represent negative and positive correlations, respectively.

A Spearman correlation analysis was performed on metabolites selected for significance from ANOVA, from which a correlogram (Figure 6A) was constructed. Interestingly, phosphocholine showed significant correlations with all metabolites. Subsequently, a network (Figure 6B) was generated using more stringent thresholds of r ≥ 0.7 and *p* ≤ 0.01, in which phosphocholine occupied a central position, connecting different classes of metabolites in a complex way. Phosphocholine displayed negative correlations with inositols, all STLs (the latter two classes being positively correlated with each other), specifically with Asn, His, Ile and Glu (these three positively correlated with each other, forming a positive-correlation subnetwork), as well as with quinic acid (which, in turn, was negatively correlated with Thr, succinic acid and sucrose). In contrast, phosphocholine was positively correlated with sucrose, succinic acid, Thr and Ala, the latter showing strong negative correlations with lactucins and inositols, forming a negative-correlation subnetwork.

## 3. Discussion

To contextualize the results, this discussion prioritizes a comparative analysis with literature centered on NMR spectroscopy of *C. intybus*. However, given constraints of available NMR datasets, comparisons are broadened to include other analytical methodologies and/or data from other *Asteraceae* species. Moreover, quantitative comparisons are based on converting our data, when possible, to those reported in the literature.

### 3.1. Qualitative Aspects: Spectral Assignment and Focus on STL

Overall, NMR studies on *C. intybus* germplasm have been performed on wild chicory (*C. intybus* subsp. *intybus*) leaves [22] and on cultivated biotypes, including Witloof leaves [23], Catalogna stems [18] and leaves [16], and Radicchio leaves [24], with the latter almost exclusively covered by HPLC-MS. To date, Sugarloaf profiles have been available by HPLC targeted to sesquiterpene lactones, hydroxycinnamic acid, flavonoids and anthocyanins [4] and fructans [25]. From the above cited literature, NMR spectra assigned a vast range of hydro-soluble metabolites, including 11 amino acids (Ala, Asp, GABA, Glu, Gln, Iso, Leu, Phe, Thr, Tyr, and Val), 7 carbohydrates (α/β-glucose, sucrose, α/β-fructofuranose, β-fructopyranose, and inulin), 4 organic acids (malic, tartaric, fumaric, and formic acids), 3 phenolics (chicoric, caftaric, and chlorogenic acids), and others (uridine, deoxyadenosine, and trigonelline); 10 liposoluble compounds (phytosterols, fatty acids, triterpenes, pigments) were also assigned [22].

In this work, the analysis expanded the leaf profiles by resolving Arg, His, phosphocholine, three sesquiterpene lactones, 8-DEOLCT-OX15, LCT-OX15, and LCP-OX15, and inulin fructose number. As for hydroxycinnamic acids, chicoric, monocaffeoyl−tartaric and chlorogenic acids normally occur in Sugarloaf leaves by HPLC [4] and in Catalogna biotype leaves by NMR [16]; here, chicoric, monocaffeoyl-tartaric acids were assigned, but the very low levels could not guarantee robust quantification data. The reasons may stand either in the need of refining extraction procedures tailored to hydroxycinnamic acids or in a constitutive low level due to genotype, environmental or cultivation technique conditions.

As for sesquiterpene lactones structure, apart from lactucin, 8-deoxylactucin and lactucopicrin, the corresponding 11(S),13-dihydro derivatives of all three compounds are also found in leaf-chicory biotypes by HPLC [4] or by combined Orbitrap-HRMS-MS/MS [26]. The ^1^H NMR signals of 11(S),13-dihydro derivatives of STLs can be obscured by the signals of other components in the aqueous extracts due to significant overlap. Consequently, while their presence cannot be ruled out, their analysis necessitates additional isolation and purification steps, which were not the objective of the present study. Chicory roots accumulate 8-DEOLCT, LCT, and LCP predominantly in oxalated forms in the latex [27]. The instability of sesquiterpene lactone conjugated forms after treatment with acidified solvents or enzymes was dealt with [19], and as a consequence, consistently, oxalyl functionalities can undergo chemical hydrolysis [25], following the addition of a water molecule under different pH conditions. Moreover, enzymatic hydrolysis cannot be excluded during the extraction, though methods of sampling (liquid nitrogen and freeze-dry) should minimize, if not eliminate, the risk. Considering that the extraction time was short and carried out under neutral pH conditions, significant degradation of conjugated STLs during sample preparation can be reasonably excluded. Moreover, both WW and WD samples were subjected to identical extraction and analytical procedures; therefore, the observed differences in STL concentrations reflect genuine differences in their native (non-degraded) content rather than preparation-related artifacts. No matrix-related effects influencing STL stability or extraction efficiency have been reported in the literature so far. Metabolic profiling of *C. intybus* roots has recently relied on Witloof chicory by LC-MS to target inulin/carbohydrates, phenolics and STLs [17], and the NMR study [22] in wild chicory roots served as a comparative baseline to confirm that, here, the "parterre" was expanded in roots by resolving STLs, polyols, organic acids (quinic and tartaric acids) and others (Arg, His, and phosphocholine).

### 3.2. Quantitative Aspects: Content Ranges

#### 3.2.1. Leaves

Quantitative ranges in leaves from this study were compared with those reported for Catalogna stems [18] and wild chicory leaves [22]. The other NMR studies were excluded as they reported relative molecular abundances or fold changes or lacked details on the extracted mass basis. Sesquiterpene lactone and inulin contents were benchmarked against established HPLC literature, prioritizing Sugarloaf chicory data. In Sugarloaf leaves, fructose accumulation exceeded Catalogna stem values by over 3-fold (118.0 vs. 25–31 mg/g DW), while glucose and sucrose levels were comparable, while the organic acid profile (malic, tartaric, quinic acids) was 2-to-3-fold lower in Sugarloaf leaves than in Catalogna stems, reflecting different physiological roles (e.g., storage versus elongating organs). Compared to wild chicory leaves, cultivated Sugarloaf biotype had a massive increase in glucose (1854 vs. 16.6 mg/100 g FW) and reduction in citric acid (31.2 vs. 128.0 mg/100 g FW), supporting the shift from an acidic/bitter wild phenotype to a sweet, domesticated leaf type. Finally, the accumulation of nitrogen-storage glutamic acid and asparagine in Sugarloaf leaves, which was higher than in wild chicory leaves [22] and Catalogna stems [18], together with the high DW (18.2%), further point at these as high-density storage organs. As for sesquiterpene lactones, NMR profiles yielded values of LCT-OX15 and LCP-OX15 similar to HPLC data for non-oxalated forms in Sugarloaf [4]: 240 and 246 mg/kg DW vs ≈211 and ≈254 mg/kg DW, respectively. In contrast, 8-deoxylactucin-oxalate by NMR was 250 vs 361 mg/kg DW, indicating a downward shift within the same magnitude order. The difference with respect to total STL content reported by Ferioli (1704 mg/kg DW) [4] is explained by the fact that six forms, including dihydro-derivatives, were quantified. Here, free (non-conjugated) STLs were not detected in freshly extracted samples, which was supported by spiking experiments with authentic standards. The presence of free STLs reported by Ferioli [4] can be attributed to the extraction by an acidified MeOH/H_2_O mixture, which must have promoted hydrolysis of oxalate-conjugated forms.

#### 3.2.2. Roots

Referring to work on hydro-soluble fraction quantification by NMR [22], a comparison of the same metabolites (after standardizing our concentrations to mg/100 g FW) revealed that while the ranges for certain compounds such as fumarate (0.9 vs. 0.5 mg/100 g) and uracil (1.7 vs. 0.6–1.4 mg/100 g) were similar, robust differences were observed for several other compounds, explained by the fact that Sugarloaf roots derive from a “high-storage” phenotype, adapted to field cultivation and production compared to wild chicory grown in pots/growth chamber. Briefly, in the Sugarloaf dataset, amino acids involved in central metabolism and protein synthesis were much more abundant, including Ala (2.6–4.6 vs. 0.06–0.36 mg/100 g) and Val (6.5–6.7 vs. 0.48–0.64 mg/100 g), inulin levels (9650–10,000 vs. 300 mg/100 g, the latter reported as amylose) and sucrose (913–1082 vs. 221–248 mg/100 g), which are values consistent with HPLC-based contents in food or industrial taproots [28]. Nitrogen metabolism also showed a functional divide, as supported by high levels of the stress-marker GABA in wild chicory, 4.2–4.4 vs. 0.7–1.2 mg/100 g FW, versus a massive accumulation of Arg (~220 mg/100 g FW) and Gln in Sugarloaf cultivated roots (60–62 vs. 8–12 mg/100 g FW). Moreover, the high Arg levels (13.21 mg/g DW) in Sugarloaf roots, ca. 3.5-fold higher than Gln and Asn, point to this residue as an important soluble nitrogen metabolism/reserve in chicory roots, as shown in other species [29]. These profiles may well reflect a cultivated root metabolically selected for efficient nutrient stockpiling versus a wild phenotype adapted for environmental stress.

The inulin content by NMR (~550–570 mg/g DW) is consistent with data obtained via enzymatic and chromatographic techniques, exceeding the ~40% DW recently reported for Sugarloaf [25]. Through ^1^H-NMR integration, the average degree of polymerization (DP) was estimated at 7.33–7.74 fructose units, consistent with the typical seasonal decline from 60 to 8–12 units between early biosynthesis and late autumn harvest. This decline reflects fructan exohydrolase activity, which hydrolyzes long-chain inulins into cryoprotective oligomers (DP 3–10) and free sugars [30]. The observed DP of ~7.5 confirms this physiological state and aligns with Witloof field root data [17]. NMR-based metabolic profiling enabled rapid inulin quantification by integrating terminal glucose and internal fructose signals, avoiding the purification/derivatization steps required by other techniques.

STLs reported in *C. intybus* roots include 8-deoxylactucin, lactucin, and lactucopicrin alongside their dihydro-derivatives, glucosides [17,19,31,32], and notably, oxalate-conjugated storage forms dominant in root latex [27]. Total root STLs typically range from 1.1 to 8.1 mg/g DW; however, reported values differ markedly by material and analytical scope. HPLC methods often target the free aglycone pool (µg/g DW range), whereas datasets capturing the broader conjugate pool in intact field roots reach mg/g DW levels [32,33]. Such divergences likely reflect differences in plant material (field-grown Sugarloaf vs. processed/transformed roots) and methodology (free aglycones vs. total conjugates). In this work, mature Sugarloaf roots contained mean LCT-OX15 and LCP-OX15 values of 714 and 344 µg/g FW, respectively, 6-to-18-fold higher than LC-MS values reported for potted Witloof roots [27]. When compared to field-grown Witloof roots containing 100-120 µg/g DW LCT and 60–70 µg/g DW LCP [17], our values for oxalate forms were 20–40 times higher (LCT-OX15 ~1960 and LCP-OX15 ~4000 µg/g DW). Crucially, Van Arkel et al. [17] quantified only free aglycones, noting that oxalated conjugates (reported only as relative peak areas) constituted the vast majority of the STL pool. Our NMR analysis enabled direct quantification of these abundant conjugate forms, yielding total STL values (~11 mg/g DW) consistent with the high storage capacity of field-grown Sugarloaf roots and comparable to industrial chicory varieties. Notably, field-grown Radicchio roots [31] show closer agreement with our Sugarloaf data, with total STLs ranging from 800 to 1000 µg/g FW from harvest to post-harvest stages (8-deoxylactucin 400–550 µg/g FW; 11,13-dihydrolactucin 200–300 µg/g FW), and contents in both field-grown bio types share similar STL accumulation distinct from Witloof. 

#### 3.2.3. Organ Valorization

From a nutritional standpoint, Sugarloaf leaves are oriented toward carbohydrate accumulation (glucose, fructose, and sucrose) and organic acids (notably malic and tartaric acids), with only modest levels of several amino acids compared with the large nitrogen pool observed in roots (e.g., arginine). This profile supports the edible head as a sweet, consumer-acceptable product, though it may be less distinctive than other leaf-chicory biotypes (Radicchio or Catalogna) in terms of specific phytochemical enrichment. The perception of bitterness in chicory is influenced by sesquiterpene lactone and hydroxycinnamic acid contents and ratios, modulated by sweetness [34]. Sugarloaf, Radicchio [31], and Witloof leaves [17] exhibit a common sugar hierarchy (fructose > glucose > sucrose). Quantitatively, the total soluble sugars in Sugarloaf (here, ca. 240 mg/g DW) align with the 300–400 mg/g DW range reported for Witloof [17]. These levels exceed Radicchio values reported as 70 mg/g DW [28] and are comparable to estimates (converted based on ca. 10% DW/FW) of 150–300 mg/g DW from other studies [31]. These variations reflect genotypic and cultivation differences; the high sugar accumulation in Sugarloaf is sensorially critical to counterbalance the bitterness of organic acids and sesquiterpene lactones, contributing to its characteristic sweet flavor.

Among the bioactive compounds detected, inulin, sesquiterpene lactones, and arginine emerged as the most distinctive, showing marked root-specific accumulation patterns. Differences between leaves and roots reflect their distinct organ functions: leaves serve primarily as photosynthetic and consumer-facing tissue, while roots function as storage and defense sinks. Roots accumulated inulin (566.7 ± 36.8 mg/g dry weight) while free fructose was undetected (likely below NMR detection limits), supporting the intense conversion of imported sugars into fructan reserves rather than retention as free hexose. Higher sesquiterpene lactone contents in roots than leaves are typical for leaf-chicory [33]; however, the striking enrichment in sesquiterpene lactone oxalates in Sugarloaf roots, 8-deoxylactucin > lactucopicrin > lactucin (5.32 ± 0.63; 4.07 ± 0.30; 1.96 ± 0.38 mg/g DW) emerges as a valorization target beyond fructan recovery, for potential bioactivity-oriented exploitation given reported therapeutic effects [11]. Finally, the exceptionally large arginine pool, 33-fold greater in roots than in leaves (13.2 ± 1.2 vs. 0.40 ± 0.06 mg/g DW), is noteworthy given the role of arginine in cardiovascular and immunological properties, reinforcing that Sugarloaf roots merit consideration for added-value exploitation.

### 3.3. Metabolic Shifts and Stress Indicators in Sugarloaf Roots Responding to Reduced Irrigation

Root metabolic characterization was prioritized (over leaf) to capture biochemical adjustments in the organ directly sensing soil water availability. While acknowledging whole-plant signaling complexity, this NMR-focused survey on novel chicory crops provides a baseline to unravel root-specific responses to reduced irrigation [12]. Overall, metabolic profiling indicated adaptive responses involving coordinated shifts in carbon and nitrogen metabolism. Although proline levels (a typically shoot water stress marker) remain below detection, the accumulation of stress-responsive AA, specifically Ala (+79%) and Thr (+17%), aligns with drought responses in roots where these function as alternative osmolytes [35]. Concurrently, the depletion of Asn and Glu suggests nitrogen flux redirection toward these drought-responsive residues, likely impacting the tricarboxylic acid cycle and contributing to succinic acid accumulation. Moreover, increased sucrose likely aids osmotic adjustment, possibly derived from partial inulin catabolism (suggested by a non-significant reduction in fructose units).

Network analysis pointed to phosphocholine as an orchestrator of these adjustments (Figure 6B), showing positive correlation with the stress-responsive cluster (sucrose, succinic acid, threonine, and alanine) and negative correlation with inositols. The coordinated depletion of myo-, chiro-, and scyllo-inositols, concomitant with reduced free choline and phosphocholine accumulation, reflects an early metabolic response consistent with rapid phosphatidylcholine turnover and intensified Kennedy pathway flux [36]. Simultaneously, increased myo-inositol consumption for stress-signaling phospholipids likely drains derived chiro- and scyllo-inositol pools [37].

The marked decline in root STLs suggests carbon reallocation from secondary metabolism toward osmolyte accumulation, hypothesized as a shift from the energy-demanding plastidial MEP pathway to primary metabolism. This is supported by the STL-osmolyte anticorrelation (Ala, Thr, and sucrose) in network analysis (Figure 6B) together with the parallel drop in STL relative content and increase in sucrose, Ala and Thr levels. While precursor/catabolite data or transcriptomics would be needed to distinguish biosynthesis vs. degradation, this pattern aligns with resource allocation theory, favoring survival under water deficit. Contrary to expectations of secondary metabolite accumulation under stress, scarce data exist on STL responses to water deficit in leafy Asteraceae. The observed drop suggests STLs serve more in biotic defense than osmotic regulation. Consistent with broad amino acid increases under root drought [35], the parallel depletion of growth-related amino acids (Glu, Ile, and Asn) further indicates pathway prioritization toward osmotic adjustment.

Collectively, these root-distinctive metabolic signatures pave the way for holistic investigation mapping root–leaf biochemical crosstalk under water deficit and evaluating bio-inoculant modulation of these trade-offs in sustainable systems [38].

## 4. Materials and Methods

### 4.1. Chemical and Reagents

Deuterated water (D_2_O, 99.97 atom% deuterium), deuterated methanol (CD_3_OD, 99.80% deuterium), and 3-(trimethylsilyl)-propionic-2,2,3,3-d4 acid sodium salt (TSP) were purchased from Euriso-Top (Saclay, France). Lactucin, 8-deoxy-lactucin and lactucopicrin were purchased from Extrasynthese (Lyon, France).

### 4.2. Sugarloaf Growth Conditions and Sampling

The experimental trials were carried out at the organic farm BioCaramadre near Fiumicino (Rome, Italy; 41.82215° N, 12.24368° E; 34 ± 5 m a.s.l.). Coated seeds of Sugarloaf chicory “Vespero” F1 (E05P.108)^P^ supplied by Enza Zaden (https://www.enzazaden.com/it accessed on 18 February 2026) were sown on 22 August 2023 into polystyrene plug trays with 170 wells (7 cm^3^ per well) with peat-based substrate (Potgrond H; Klasmann-Deilmann GmbH, Geeste, Germany), germinated in semi-protected tunnels with daily irrigation to field capacity and controlled temperature (5 °C below ambient); no pesticides were applied.

Three-to-four-leaf seedlings were transplanted on 19 September 2023 into soil pre-fertilized with organic manure (Fertildung^®^, NPK 3–3–2, Fomet S.p.A., San Pietro di Morubio, VR, Italy) and potassium–magnesium sulfate (Patentkali^®^, K+S Minerals and Agriculture GmbH, Kassel, Germany; 8 q ha^−1^). No chemical pest or disease control was required. Irrigation was applied via Netafim Meganet™ (Netafim Ltd., Tel Aviv, Israel) sprinklers (10 × 12 m plot) at two flow rates, 55 and 35 L h^−1^, supplying 100% (W100) and 67% (W67) of full restitution, respectively. Following an initial 3 h irrigation (55 L h^−1^) to minimize transplant shock, experimental irrigations occurred on 13 October, 17 November, and 11 December 2023 (70 min duration; 5 and 3 mm for W100 and W67, respectively). Soil tension measurements on irrigation dates indicated mild stress in W100 (−70, −30, −30 mbar) and severe stress in W67 (−100 mbar on all dates). Plants were harvested on 19 December 2023.

### 4.3. Experimental Design, Sampling Procedures and Storing

The experiment followed a completely randomized design with two irrigation regimes: W100 (control) and W67 (reduced irrigation). Treatments were randomly assigned within a uniform 500 m^2^ area. Each treatment comprised three plots (1.2 m × 8 m, density ca. 6.5 plants m^−2^) corresponding to 173 plants per treatment. In this design, plots were considered experimental units (three plots per treatment), whereas plants or pooled tissues from plants within each plot constituted the biological samples for analysis. Regarding root sampling, 5 plants were randomly uprooted from each experimental plot (replicate) and immediately transported to the laboratory under cool, dark conditions. Roots were manually separated from heads, gently washed to remove adhering soil, and pooled by plot (five roots per plot) to obtain three biological replicate batches per treatment. Pooled roots were snap-frozen in liquid nitrogen, ground to a fine powder, stored at −80 °C, and subsequently freeze-dried following the procedure previously described [18]. Additional samples for tissue analysis (roots and leaves) were collected from a separate set of plots within the same area, grown under full irrigation and harvested on 21 December 2023. Heads were cleaned to commercial standards and longitudinally quartered; one quarter per head was sampled to represent the edible portion. Ten leaf quarters from each plot were pooled to generate three biological replicate leaf batches, and roots from the same plants were sampled as described above.

### 4.4. Tissue Extraction and Sample Preparation for NMR Analyses

The extraction protocol was the same for leaves and roots. Each extraction was performed using 50 mg of lyophilized and finely ground tissue and 1.0 mL of H_2_O/ACN (1:1 *v*/*v*) mixture. Samples were then sonicated for one minute, vortexed and centrifuged for three minutes at 12,000 rpm. The resulting supernatants were collected into a 20 mL glass vial and dried under N_2_ flux at room temperature. Dried samples were dissolved in 0.7 mL of 400 mM water phosphate buffer at pH = 7.0 (containing 1 mM TSP) and put into a standard 5 mm NMR glass tube. Three replicates of each tissue were extracted and analyzed.

### 4.5. Solid-State Extraction and Fractionation of Sesquiterpene Lactones (STLs)

To identify the STLs present in extracts, SPE separation was performed. Since STL amounts were quite low, 400 mg of lyophilized root sample and 4 mL of the H_2_O:ACN (1:1) mixture were used for extraction. The dried extract was dissolved in 2 mL of pure water. A 30 mg Waters Oasis HLB cartridge was employed for SPE separation. It contains hydrophilic–lipophilic-balanced, water-wettable, reversed-phase sorbent made from a specific ratio of two monomers, the hydrophilic N-vinylpyrrolidone and the lipophilic divinylbenzene. Two milliliters of methanol were used for the solid-phase activation step, and two milliliters of pure water were used for the conditioning step. The entire sample (2 mL) was passed through the cartridge for loading, and another 2 mL of pure water was used for washing away hydrophilic metabolites (sugars, amino acids, organic acids). Elution was performed using 2 mL of H_2_O: MeOH (8:2 *v*/*v*) for collecting the first fraction, and consecutively, 2 mL of H_2_O: MeOH (1:1 *v*/*v*) for the second fraction; finally, 2 mL of 100% MeOH was used for the last fraction. All collected fractions were dried under N_2_ flux. Each sample was then dissolved in 0.7 mL of CD_3_OD or 0.7 mL of buffered D_2_O (pH = 7.0) for NMR characterization.

### 4.6. NMR Analysis and Metabolites Characterization

All NMR spectra were recorded using a 600 MHz Bruker Avance III HD spectrometer (Bruker-Milan, Italy) equipped with a BBI inverse probe. TopSpin 3.6 Bruker software was used for spectral recording and spectral processing. ^1^H NMR spectra were recorded with 200 transients, 7 s relaxation delay, 90° pulse at 11.5 µs, 32K size of time domain, and 12.0 ppm spectral width. Presaturation was used to suppress the strong residual HDO signal. Spectra were processed using an exponential multiplication factor of 0.3 Hz before Fourier transformation, followed by manual phase and baseline corrections. The TSP signal (δ = 0.00 ppm) and CD_2_HOD signal (δ = 3.31 ppm) of partially deuterated methanol were the chemical shift references for buffered D_2_O and deuterated methanol solutions, respectively. A series of 2D-NMR (^1^H-^1^H TOCSY, ^1^H-^13^C HSQC, and ^1^H-^13^C HMBC) spectra was also recorded to assign ^1^H and ^13^C spectra and identify metabolites present in the entire aqueous extracts and in SPE fractions containing sesquiterpene lactones. The mixing time was set to 80 ms in TOCSY, the coupling constant *J*^1^_C–H_ was 150 Hz in HSQC, and a delay of 80 ms was used for the evolution of long-range couplings in HMBC. A DOSY experiment was performed using a bipolar LED sequence with a sine-shaped gradient of different intensities and the following parameters: diffusion time of 100 ms, gradient duration δ/2 was 1.5 ms. The gradient strength was incremented in 32 steps from 2% up to 95% of the maximum gradient strength (55.25 G/cm).

### 4.7. NMR Data Quantification and Statistical Analysis

Quantitative analysis of identified metabolites was executed by integrating selected characteristic signals (Supplementary Table S1). TSP was used as an internal standard for quantification and as a reference for spectral calibration. ANOVA was performed using R (version 4.3.1) within the RStudio software (version 2023.06.1). Metabolite–metabolite associations were evaluated using Spearman (Rank) correlation. Significance was adjusted for multiple testing using the Benjamini–Hochberg FDR procedure. Correlation network analysis was performed using Hmisc and qgraph R packages. Pairwise Spearman correlations and associated p-values were computed using rcorr, and p-values were adjusted by the Benjamini–Hochberg false discovery rate (FDR) procedure. Correlations with |r| ≥ 0.70 and FDR ≤ 0.01 were visualized, with edge width proportional to |r| and colors indicating the sign of the correlation.

## 5. Conclusions

The comparative NMR profiling of ‘Sugarloaf’ expands known phytochemical ranges, revealing that leaves have a carbohydrate-rich profile (high hexoses/sucrose, ~4.5% DW), while roots function as massive storage sinks for inulin (~57% DW) and sesquiterpene lactones (STLs, ~11 mg/g DW), highlighting their potential for valorization beyond waste biomass. NMR analysis, combined with optimized extraction protocols and spectra refinements, enabled the confident spectral assignment of lactucin, 8-deoxylactucin, and lactucopicrin in oxalate-conjugated forms, providing a robust root metabolome profile that expands known phytochemical ranges beyond LC-MS datasets. Under water depletion, roots displayed a distinct metabolic reprogramming strategy, relying on the osmotic adjustment via accumulation of alanine (+79%) and threonine (+17%) alongside sucrose (+19%). Network analysis identified phosphocholine as a central node coordinating membrane lipid turnover with the depletion of inositols. Crucially, the marked decline in root STLs due to reduced irrigation raises questions about the “stress-defense” paradigm. This suggests a strategic trade-off where carbon flux is diverted from the energy-demanding plastidial MEP pathway (responsible for sesquiterpene lactone synthesis) to prioritize glycolysis-derived survival metabolites (alanine) and ATP generation. These findings establish a baseline for root-specific stress adaptation in chicory, supporting a prioritization of primary survival pathways over secondary defense under water deficit, and pave the way for studies on root–leaf crosstalk and bio-inoculant applications in sustainable cropping.

## Figures and Tables

**Table 1:** Water-soluble metabolites identified in Sugarloaf chicory leaf (L) and root (R) extracts.

Compound	Assignment	^1^H (ppm)	J^1^_H-H_ [(Hz)]	^13^C (ppm)	Tissue
*Amino acids*					
Alanine	β−CH_3_	1.48	d (7.2)	17.1	L, R
	α−CH	3.79	m	51.4	
Arginine	β–CH_2_	1.67; 1.74	m	24.8	L, R
	γ–CH_2_	1.93	m	28.6	
	δ–CH_2_	3.24	m	41.4	
	α−CH	3.78	m	55.2	
Aspartic acid	β−CH	2.79	dd (17.4; 3.90)	37.5	L, R
	β′−CH	2.71	dd (17.40; 8.32)	37.5	
	α−CH	3.94	m	53.1	
Asparagine	β−CH	2.94	dd (16.88; 4.48)	35.6	L, R
	β′–CH	2.89	dd (16.88; 7.26)	35.6	
	α–CH	4.00	m	52.1	
GABA	β–CH_2_	1.90	m	24.8	L, R
	α–CH_2_	2.30	t (7.4)	35.3	
	γ–CH_2_	3.01	t (7.6)	40.1	
Glycine	α–CH_2_	3.57	s	42.4	L
Glutamic acid	β−CH_2_	2.07; 2.13	m	27.9	L, R
	γ–CH	2.35	m	34.4	
	α–CH	3.76	m	55.5	
Glutamine	β−CH_2_	2.15	m	27.2	L, R
	γ–CH	2.50	m	32.5	
	α–CH	3.77	m	55.5	
Histidine	β−CH_2_	3.28; 3.21	m	28.6	L, R
	α–CH	4.01	dd	55.5	
	CH-3	6.89	s	118.1	
	CH-4	7.78	s	136.7	
	CH-5	-	-	131.0	
Isoleucine	δ−CH_3_	0.95	m	14.5	L, R
	γ−CH_3_	1.015	d (7.0)	15.7	
	α−CH	3.67	m	60.4	
Leucine	–CH_3_	0.961	m	22.0	L, R
	–CH_3_	0.968	m	22.9	
	γ−CH	1.73	m	24.8	
	β−CH_2_	1.74	m	40.9	
	α−CH	3.74	m	54.3	
Lysine	γ–CH_2_	1.48	m	22.4	L, R
	δ–CH_2_	1.73	m	27.4	
	β–CH_2_	1.91	m	30.9	
	ε–CH_2_	3.03	t	40.0	
	α–CH	3.77	t	55.4	
Phenylalanine	β−CH_2_	3.28; 3.15	m	37.4	L, R
	α–CH	4.00	m	56.9	
	CH-4	7.39	m	128.3	
	CH-3,5	7.43	m	130.1	
	CH-2,6	7.33	m	130.3	
Pyroglutamic acid	γ−CH_2_	2.40	m	30.8	L
	β−CH_2_	2.50; 2.03	m	26.3	
	α−CH	4.18	dd	59.3	
Threonine	γ−CH_3_	1.33	d (6.9)	20.3	L, R
	β−CH	3.59	-	61.7	
	α−CH	4.25	dd (6.5; 4.9)	66.9	
Tyrosine	β−CH_2_	2.86; 2.71	-	41.0	L, R
	α−CH	3.44	-	58.9	
	CH-3,5	6.90	d	119.8	
	CH-2,6	7.12	d	132.0	
	CH-1	-	-	128.0	
	CH-4	-	-	-	
Valine	−CH_3_	0.99	d (6.9)	17.5	L, R
	−CH_3_’	1.04	d (6.9)	18.9	
	β−CH	2.27	dd (4.3; 6.9)	30.1	
	α−CH	3.60	m	61.3	
*Amino Alcohols*					
Choline	−N(CH_3_)_3_	3.20	s	54.9	L, R
	−NCH_2_	3.52	-	68.3	
	−CH_2_(OH)	4.08	-	55.9	
Phosphocholine	−N(CH_3_)_3_	3.22	s	55.0	L, R
	−NCH_2_^-^	3.60	-	67.4	
	−CH_2(_OPO_3_^-^)	4.17	-	59.0	
Ethanolamine	−CH_2_(NH_2_)	3.14	m	42.2	L, R
	−CH_2_(OH)	3.83	-	58.6	
*Organic acids*					
Citric acid	CH_2_	2.67; 2.54	d (15.3)	46.4	L, R
Formic acid	HCOOH	8.46	s	173.9	L, R
Fumaric acid	−C=CH	6.52	s	135.9	L, R
Lactic acid	−CH_3_	1.33	d (6.5)	21.1	L, R
	CH(OH)	4.11	q (6.5)	69.5	
Malic acid	CH_2_	2.36; 2.66	m	43.5	L, R
	CH(OH)	4.29	dd (10.1; 3.1)	71.3	
Quinic acid	C-1	-		78.1	
	CH_2_-2	2.04; 1.97	-	38.4	L, R
	CH(OH)-3	3.55	-	76.3	
	CH(OH)-4	4.02	-	67.9	
	CH(OH)-5	4.15	-	71.4	
	CH_2_-6	2.06; 1.87	-	41.7	
	COOH	-		182.2	
Succinic acid	−CH_2_−	2.41	s	35.1	L, R
	−COOH	-	-	180.3	
Tartaric acid	CH(OH)	4.34	s	74.9	L, R
*Sugars*					
β-Glucose	−CH-1	4.66	d (7.9)	96.9	L, R
	−CH-2	3.26	dd (9.4; 8.0)	75.1	
	−CH-3	3.48	-	76.8	
	−CH-4	3.41	-	70.6	
	−CH-5	3.48	-	76.9	
	−CH-6	3.90; 3.74	-	61.8	
α-Glucose	−CH-1	5.24	d (3.8)	93.1	L, R
	−CH-2	3.55	-	72.3	
	−CH-3	3.73	-	73.7	
	−CH-4	3.42	-	70.7	
	−CH-5	3.84	-	72.6	
	−CH-6	3.85; 3.77	-	61.7	
β-Galactose	−CH-1	4.59	d (7.9)	97.4	L
	−CH-2	3.50	-	72.8	
	−CH-3	3.66	-	73.7	
	−CH-4	3.94	-	69.7	
	−CH-5	4.08	-	71.4	
	−CH-6	3.76	-	62.0	
α-Galactose	−CH-1	5.27	d (3.8)	93.3	L
	−CH-2	3.82	-	69.3	
	−CH-3	3.85	-	70.2	
	−CH-4	4.00	-	70.2	
	−CH-5	4.08	-	71.4	
	−CH-6	3.70	-	76.0	
β-D-Fructofuranose	−CH-1,1’	3.60; 3.57	-	63.7	L
	−C-2	-	-	102.4	
	−CH-3	4.11	m	76.4	
	−CH-4	4.12	-	75.4	
	−CH-5	3.83	-	81.6	
	−CH-6,6’	3.81; 3.68	-	63.3	
α-D-Fructofuranose	−CH-3	4.12	-	82.9	L
	−CH-5	4.06	-	82.3	
β-D-Fructopyranose	−CH-1,1’	3.72;3.57	-	64.7	L
	−CH-3	3.81	-	68.5	
	−CH-4	3.90	-	70.7	
	−CH-5	4.00	-	70.1	
	−CH-6	4.03; 3.79	-	64.3	
Sucrose	−CH-1(Glc)	5.42	d (3.8)	93.2	L, R
	−CH-2	3.56	-	72.0	
	−CH-3	3.77	-	73.5	
	−CH-4	3.48	-	70.2	
	−CH-5	3.85	-	73.3	
	−CH-6	3.82	-	61.1	
	−CH-1(Fru)	3.68	-	62.3	
	−C-2	-	-	104.7	
	−CH-3	4.22	-	77.4	
	−CH-4	4.06	-	75.0	
	−CH-5	3.91	-	82.4	
	−CH-6	3.83	-	63.3	
Raffinose	Gal-1	5.00	-	99.4	L
	Gal-2	3.83	-	69.3	
	Gal-3	3.91	-	70.3	
	Gal-4	4.01	-	70.1	
	Gal-5	3.96	-	71.9	
	Gal-6	3.76	-	62.0	
	Glu-1	5.43	-	93.0	
	Glu-2	3.58	-	71.8	
	Glu-3	3.77	-	73.5	
	Glu-4	3.55	-	70.3	
	Glu-5	4.06	-	72.3	
	Glu-6	4.05; 3.70	-	66.7	
	Fru-1	3.68	-	72.3	
	Fru-2	-	-	104.70	
	Fru-3	4.22	-	77.2	
	Fru-4	4.07	-	74.9	
	Fru-5	3.90	-	82.2	
	Fru-6	3.81	-	63.4	
Inulin	Glu-1	5.44	-	93.4	R
	Glu-2	3.58	-	71.9	
	Glu-3	3.77	-	73.5	
	Glu-4	3.55	-	70.3	
	Glu-5	3.85	-	73.4	
	Glu-6	3.83	-	61.1	
	Fru-1	3.92; 3.87	-	72.3	
	Fru-2	-	-	104.09	
	Fru-3a	4.21	-	77.8	
	Fru-3b	4.25	-	78.3	
	Fru-3c	4.28	-	77.9	
	Fru-4a	4.06	-	75.0	
	Fru-4b	4.11	-	75.4	
	Fru-5	3.89	-	82.2	
	Fru-6	3.83	-	63.3	
*Sugar Alcohols*			-		
Myo-inositol	−CH-1	4.07	-	73.2	L, R
	−CH-2,5	3.54	-	71.9	
	−CH-3,6	3.63	-	73.2	
	−CH-4	3.29	t (9.36)	75.2	
Chiro-inositol	−CH-1,6	4.05	-	72.7	L, R
	−CH-2,5	3.76	-	71.4	
	−CH-3,4	3.59	t	73.8	
Scyllo-inositol	−CH(OH)	3.35	s	74.4	L, R
*Miscellaneous*					
Trigonelline	−N−CH_3_	4.44	s	49.3	L
	−C=CH-5	8.08	t (6.6)	128.7	
	−C=CH-4	8.84	dd (7.2; 1.2)	145.8	
	−C=CH-6	8.84	dd (7.2; 1.2)	147.1	
	−CH-2	9.12	s	146.9	
Chicoric acid	CH(O)COOH	5.54	s	75.6	L
	=CH−COOH	6.47	d (16.0)	115.2	
	−CH=	7.73	d (16.0)	147.7	
	CH-2’	7.26	m	116.2	
	CH-5’	6.97	m	117.3	
	CH-6’	7.17	m	123.8	
Monocaffeoyltartaric acid	CH(O)COOH	5.30	d (2.2)	77.7	L
	CH(OH)COOH	4.55	d (2.2)	73.4	
	=CH−COOH	6.43	d (16.0)	115.4	
	−CH=	7.67	d (16.0)		
Uridine	−CH_2_-5	3.93; 3.82	-	61.1	L, R
	−CH-4	4.24	-	70.5	
	−CH-3	4.36	-	74.9	
	−CH-2	4.15	-	85.2	
	−CH-1	5.91	d	10.3.5	
	−C=C–H-7	5.93	d	90.4	
	−C=C−H-6	7.90	-	143.0	

^1^H/^13^C: proton/carbon chemical shifts (ppm); J (Hz): coupling constant showing proton connectivity; m: multiplet; d: doublet; t: triplet; dd/ddd/dt/ddt/ddq/dtd/q/s/td: complex splitting patterns; N.D.: not determined; CH_2_/CH/CH_3_/C/C(O): carbon types (methylene/methine/methyl/quaternary/carbonyl). Shifts referenced to TSP (0.0 ppm) in D_2_O; J values confirm structural connectivity; -, absent.

**Table 2:** Sugarloaf sesquiterpene lactone assignment in CD_3_OD.

Assignment	^1^H (ppm)	*J* [(Hz)]	^13^C (ppm)	^1^H (ppm) ^a^	^13^C (ppm) ^a^
	**Lactucopicrin-15-oxalate**			**Lactucopicrin**	
C-1	-	-	134.6	-	134.6
C-2	-	-	196.6	-	197.0
CH-3	6.45	s	134.5	6.40	133.6
C-4	-	-	167.9	-	176.3
CH-5	4.00	d (10.1)	49.3	3.83	49.8
CH-6	3.82	t (10.1)	82.1	3.76	82.6
CH-7	3.48	tt (10.2; 3.0)	54.9	3.41	55.5
CH(OH)-8	4.91	td (10.5;1.9)	70.7	4.88	71.2
CH_2_-9	2.87	dd (13.4; 10.8)	44.6	2.83	45.0
	2.46	dd (13.4; 2.1)		2.42	
C-10	-	-	148.1	-	148.1
C-11	-	-	137.9	-	138.1
C(O)-12	-	-	170.0	-	170.3
CH_2_-13	5.93	d (3.0)	121.8	5.90	122.3
	5.28	d (3.0)		5.23	
CH_3_-14	2.41	s	21.1	2.39	21.5
CH_2_-15	5.42	d (17.1)	64.9	4.85	63.2
	5.10	d (17.1)		4.40	
CH-4′/8′	7.14	d (8.6)	131.2	7.12	131.6
CH-5′/7′	6.76	d (8.6)	116.2	6.74	116.5
C-1′	-	-	172.2	-	172.6
CH_2_-2′	3.64	s	41.2	3.62	41.7
C-3′	-	-	125.2	-	125.8
C(OH)-6′	-	-	157.8	-	158.1
	**Lactucin-15-oxalate**			**Lactucin**	
C-1	-	-	132.1	-	139.4
C-2	-	-	197.4	-	197.3
CH-3	6.44	q (1.6)	134.3	6.40	134.0
C-4	-	-	168.2	-	168.1 ^b^
CH-5	3.95	d (10.2)	50.0	3.80	50.1
CH-6	3.74	t (10.2)	82.4	3.69	82.9
CH-7	3.15	tt (10.1; 3.1)	58.1	3.08	58.6
CH(OH)-8	3.84	m	68.4	3.82	68.6
CH_2_-9	2.89	dd (13.7; 10.8)	49.6	2.62	49.7
	2.44	m		2.41	
C-10	-	-	149.3	-	149.6
C-11	-	-	133.1	-	133.4
C(O)-12	-	-	168.8	-	176.4 ^b^
CH_2_-13	6.23	dd (3.1; 1.3)	122.5	6.22	122.9
	6.14	dd (3.1; 1.3)		6.13	
CH_3_-14	2.42	s	21.9	2.42	21.9
CH_2_-15	5.41	ddd (17.1; 1.7; 0.6)	64.4	4.88	63.2
	5.07	ddd (17.1; 1.4; 1.1)		4.42	
Oxalate (COO)			165.6		-
	**8-deoxy-lactucin-15-oxalate**			**8-deoxy-lactucin**	
C-1	-	-	132.9	-	140.7 ^c^
C-2	-	-	197.4	-	195.7
CH-3	6.44	q (1.6)	134.3	6.41	133.2
C-4	-	-	168.6	-	170.4 ^d^
CH-5	3.96	d (10.2)	51.1	3.82	51.2
CH-6	3.68	t (10.2)	85.4	3.64	85.6
CH-7	3.08	ddq (11.4; 10.1; 3.0)	53.3	3.03	53.6
CH_2_-8	2.27	ddt (13.7; 5.9; 2.5)	25.1	2.26	38.2 ^e^
	1.45	dddd (13.7; 12.7; 11.4; 1.6)		1.44	
CH_2_-9	2.65	ddd (14.5; 12.8; 1.9)	38.0	2.36	25.3 ^e^
	2.43	m		2.43	
C-10	-	-	155.9	-	155.8
C-11	-	-	140.2	-	133.1 ^c^
C(O)-12	-	-	170.7	-	176.2 ^d^
CH_2_-13	6.10	d (3.2)	118.9	6.09	119.2
	5.56	d (3.2)		5.56	
CH_3_-14	2.43	s	21.8	2.43	22.0
CH_2_-15	5.41	ddd (17.1; 1.7; 0.6)	64.4	4.88	63.1
	5.07	ddd (17.1; 1.4; 1.1)		4.42	
Oxalate (COO)	-		165.6		

^a^, data from reference [20]; ^b^, ^c^, ^d^, and ^e^, repeated letter pairs indicate the values to be swapped in the order of assignment, specifically: ^b^, C-12 δ 176.4 → 168.1 ppm (HMBC to H-13); ^c^, C-1 δ 140.7 → 133.1 ppm (HMBC to H-5); ^d^, C-4 δ 170.4 → 176.2 ppm (HMBC to H-15); ^e^, C-8 δ 38.2 → 25.3 ppm (HSQC to H-8).

**Table 3:** Sugarloaf sesquiterpenes lactone assignment in buffered D_2_O.

Assignment	^1^H (ppm)	*J* (Hz)	^13^C (ppm)	^1^H (ppm), Standard	*J* (Hz)	^13^C (ppm), Standard
	**Lactucopicrin-15-oxalate**			**Lactucopicrin**		
C-1	-	-	134.4	-		
C-2	-	-	198.3	-		
CH-3	6.48	m	134.3	6.48	m	
C-4	-	-	175.9	-		
CH-5	4.02	m	49.3	3.96	d (10.1)	
CH-6	3.90	m	N.D.	3.98	t (10.1)	
CH-7	3.55	m	54.2	3.56	m	
CH(OH)-8	4.97	m	71.1	5.02	td (10.9; 2.0)	
CH_2_-9	2.90	m	44.5	2.92	dd (13.6; 11.2)	
	2.50	m		2.50	dd (13.6; 2.0)	
C-10	-	-	148.1	-		
C-11	-	-	137.9	-		
C(O)-12	-	-	170.0	-		
CH_2_-13	6.06	d (2.2)	123.7	6.07	d (3.2)	
	5.40	N.D.		5.43	d (3.0)	
CH_3_-14	2.41	s	21.1	2.39	s	
CH_2_-15	5.43	d (17.0)	65.5	4.85	d (18.4)	
	5.17	d (17.0)		4.53	dd (18.4; 1.5)	
CH-4′/8′	7.24	d (8.5)	131.9	7.26	d (8.5)	
CH-5′/7′	6.91	d (8.5)	116.6	6.93	d (8.5)	
C-1′	-	-	N.D.	-		
CH_2_-2′	3.76	s	41.1	3.78	s	
C-3′	-	-	126.4	-		
C(OH)-6′	-	-	156.0	-		
Oxalate (COO)			N.D.			
	**Lactucin-15-oxalate**			**Lactucin**		
C-1	-	-	133.4	-	-	133.2
C-2	-	-	N.D.	-	-	199.4
CH-3	6.46	d (1.3)	132.8	6.47	m	133.2
C-4	-	-	175.6		-	176.0
CH-5	4.02	d (10.5)	49.5	3.92	m	49.4
CH-6	3.92	t (10.6)	82.4	3.92	m	82.9
CH-7	3.27	N.D.	57.1	3.24	m	57.4
CH(OH)-8	4.00	N.D.	67.7	4.01	ddd (10.8;10.1; 2.3)	67.9
CH_2_-9	2.94	N.D.	49.0	2.93	dd (13.7;10.9)	49.1
	2.52			2.51	dd (13.7; 2.3)	
C-10	-	-	152.4	-	-	151.8
C-11	-	-	137.9	-	-	137.7
C(O)-12	-	-	173.4	-	-	173.4
CH_2_-13	6.23	dd (4.9; 3.0)	124.3	6.22	t (6.3)	124.3
CH_3_-14	2.41	s	22.8	2.41	s	22.7
CH_2_-15	5.46	N.D.	65.6	4.90	dd (18.4; 1.8)	62.7
	5.19	d (16.9)		4.54	dd (18.4; 1.8)	
Oxalate (COO)			165.0			
	**8-deoxy-lactucin-15-oxalate**			**8-deoxy-lactucin**		
C-1	-	-	131.7	-	-	131.9
C-2	-	-	199.3	-	-	199.8
CH-3	6.45	d (1.3)	134.0	6.45	q (1.6)	132.8
C-4	-	-	169.0	-	-	176.0
CH-5	4.02	d (10.5)	50.8	3.92	d (10.3)	50.6
CH-6	3.85	m	85.9	3.82	t (10.3)	86.1
CH-7	3.14	m	52.7	3.11	ddq (11.3; 10.3; 3.1)	52.8
CH_2_-8	2.29	dddd (13.8; 8.7; 5.5; 2.8)	24.5	2.28	ddt (13.8; 6.3; 2.5)	24.5
	1.48			1.47	dtd (13.7; 12.1; 1.6)	
CH_2_-9	2.62	d (12.9)	38.1	2.62	ddd (14.6; 12.6; 2.0)	38.0
	2.51	d (3.1)		2.49	ddd (14.6; 6.2; 1.7)	
C-10	-	-	160.0	-	-	159.3
C-11	-	-	139.3	-	-	139.3
C(O)-12	-	-	174.0	-	-	173.8
CH_2_-13	6.17	d (3.2)	121.2	6.16	d (3.4)	121.1
	5.68	d (3.2)		5.67	d (3.4)	
CH_3_-14	2.42	s	22.8	2.42	s	22.8
CH_2_-15	5.46	dt (17.1; 1.6)	65.6	4.90	dd (18.3; 1.8)	62.6
	5.19	dd (17.1; 1.6)		4.53	d (18.3)	
Oxalate (COO)			165.0			-

Footnote legend is the same as in Table 1.

**Table 4:** Metabolite content (mg g^−1^ DW) in leaves and roots ^a^.

Metabolite	Leaves	Roots	S
*Amino acids*			
Alanine	0.77 ± 0.10	0.14 ± 0.01	***
Arginine	0.40 ± 0.06	13.21 ± 1.21	***
Asparagine	4.34 ± 0.33	3.88 ± 0.31	n.s.
Aspartic acid	1.56 ± 0.15	1.19 ± 0.02	*
GABA	0.11 ± 0.01	0.057 ± 0.018	*
Glutamine	13.75 ± 0.61	3.61 ± 0.31	***
Glutamic acid	1.90 ± 0.04	1.14 ± 0.03	***
Histidine	0.26 ± 0.10	0.25 ± 0.03	n.s.
Isoleucine	0.20 ± 0.02	0.20 ± 0.02	n.s.
Leucine	0.063 ± 0.008	0.087 ± 0.008	*
Phenylalanine	0.12 ± 0.01	0.110 ± 0.007	n.s.
Threonine	0.43 ± 0.04	0.36 ± 0.02	n.s.
Tyrosine	0.10 ± 0.02	-	
Valine	0.26 ± 0.02	0.40 ± 0.04	**
*Amino alcohols*			
Choline	0.52 ± 0.06	0.50 ± 0.02	n.s.
Phosphocholine	0.12 ± 0.01	0.032 ± 0.002	***
Ethanolamine	0.31 ± 0.02	0.35 ± 0.23	n.s.
*Organic acids*			
Citric acid	1.71 ± 0.28	4.20 ± 0.14	***
Lactic acid	0.034 ±0.008	0.080 ± 0.005	**
Formic acid	0.033 ± 0.002	0.079 ± 0.006	***
Fumaric acid	0.020 ± 0.004	0.052 ± 0.005	**
Malic acid	9.35 ± 0.75	2.83 ± 0.17	***
Quinic acid	1.90 ± 0.05	0.66 ± 0.16	***
Tartaric acid	3.29 ± 0.38	0.19 ± 0.01	***
*Carbohydrates*			
Fructose	118.03 ± 3.59	-	
β-Galactose	0.55 ± 0.03	-	
Glucose	101.62 ± 2.71	0.58 ± 0.08 (β)	***
Sucrose	25.54 ± 2.12	51.74 ± 1.46	***
Raffinose	10.63 ± 1.08	-	
Inulin	-	566.72 ± 36.80	
*Sugar alcohols*			
Chiro-inositol	0.44 ± 0.07	1.56 ± 0.15	***
Myo-inositol	1.10 ± 0.07	1.28 ± 0.20	n.s.
Scyllo-inositol	0.21 ± 0.04	0.26 ± 0.07	n.s.
*Sesquiterpene lactone oxalates*			
8-deoxy-lactucin	0.25 ±0.03	5.32 ± 0.63	***
Lactucin	0.24 ± 0.01	1.96 ± 0.38	**
Lactucopicrin	0.25 ± 0.006	4.07 ± 0.30	***
*Miscellaneous*			
Trigonelline	0.068 ± 0.007	-	
Uridine	0.15 ± 0.02	0.10 ± 0.02	*

^a^, values are reported as mean and standard deviation; S, significance by ANOVA test; n.s., non-significant; *, *p* ≤ 0.05; **, *p* ≤ 0.01; ***, *p* ≤ 0.001. Relative content change (RCC) is calculated as [(WD − WW)/WW] × 100. The mean dry weight to fresh weight ratio (DW/FW) was 18.22% and 17.56% for leaves and roots; to convert tabulated DW values to a fresh weight basis (mg g^−1^ FW), divide by 5.48 and 5.69, respectively.

**Table 5:** Root metabolite content (mg g^−1^ DW) of Sugarloaf grown under well-watered (WW) and water-deficit (WD) regimes during an autumn–winter open-field cycle (2023).

Compound	WW	WD	S	RCC
Ala	0.147 ± 0.006 b	0.263 ± 0.006 a	***	79%
Arg	12.593 ± 0.327	12.337 ± 0.617	n.s.	−2%
Asn	3.740 ± 0.106 a	2.643 ± 0.095 b	***	−29%
Asp	1.197 ± 0.023	1.127 ± 0.057	n.s.	−6%
GABA	0.067 ± 0.015	0.040 ± 0.010	n.s.	−40%
Gln	3.540 ± 0.262	3.433 ± 0.189	n.s.	−3%
Glu	1.127 ± 0.015 a	0.950 ± 0.036 b	**	−16%
His	0.237 ± 0.015 a	0.190 ± 0.020 b	*	−20%
Ile	0.190 ± 0.010 a	0.123 ± 0.006 b	**	−35%
Leu	0.083 ± 0.006	0.090 ± 0.001	n.s.	8%
Phe	0.110 ± 0.001	0.097 ± 0.006	n.s.	−12%
Thr	0.353 ± 0.012 b	0.413 ± 0.006 a	**	17%
Val	0.383 ± 0.021	0.373 ± 0.015	n.s.	−3%
TOT-AA	23.667 ± 0.725 a	22.080 ± 0.528 b	*	−7%
CA	4.143 ± 0.045	4.287 ± 0.250	n.s.	3%
FA	0.052 ± 0.005	0.052 ± 0.002	n.s.	1%
MA	2.947 ± 0.049	2.397 ± 0.376	n.s.	−19%
SA	0.357 ± 0.006b	0.423 ± 0.025 a	*	19%
TOT-TCA	7.490 ± 0.070	7.157 ± 0.650	n.s.	−4%
GLC	0.620 ± 0.056	0.817 ± 0.330	n.s.	32%
INU	549.233 ± 11.434	570.040 ± 7.214	n.s.	4%
SUC	51.950 ± 1.531 a	61.587 ± 3.465 b	*	19%
TOT-CAR	601.797 ± 12.99 a	632.440 ± 7.355 b	*	5%
INU-F	7.737 ± 0.255	7.330 ± 0.218	n.s.	−5%
CHO	0.507 ± 0.015	0.473 ± 0.015	n.s.	−7%
EthAM	0.413 ± 0.210	0.330 ± 0.020	n.s.	−20%
P-CHO	0.030 ± 0.001 b	0.040 ± 0.001a	***	33%
TOT-PP	0.950 ± 0.195	0.840 ± 0.036	n.s.	−12%
Chiro-I	1.037 ± 0.023 a	0.637 ± 0.130 b	*	−39%
Myo-I	1.177 ± 0.015 a	0.930 ± 0.046 b	**	−21%
Scyllo-I	0.260 ± 0.070	0.253 ± 0.006	n.s.	−3%
TOT-POL	2.477 ± 0.107a	1.827 ± 0.172 b	**	−26%
FOR	0.077 ± 0.006	0.077 ± 0.006	n.s.	0%
LAC	0.080 ± 0.001 a	0.070 ± 0.000b	***	−13%
QUI	0.753 ± 0.012 a	0.477 ± 0.110b	*	−37%
TAR	0.200 ± 0.000	0.167 ± 0.021	n.s.	−17%
TOT-OA	1.107 ± 0.012	0.793 ± 0.131	n.s.	−28%
8-DEOLCT-OX15	5.697 ± 0.203 a	2.807 ± 0.188 b	***	−51%
LCP-OX15	4.233 ± 0.194 a	2.543 ± 0.067 b	**	−40%
LCT-OX15	2.143 ± 0.225 a	1.130 ± 0.082 b	**	−47%
TOT-STLs	12.070 ± 0.252 a	6.480 ± 0.334 b	***	−46%
Uridine	0.097 ± 0.025	0.093 ± 0.006	n.s.	−4%

Values are reported as mean ± standard deviation. S, statistical significance was determined by one-way ANOVA, with different letters indicating significant differences; n.s. denotes non-significant results, while *, **, and *** indicate significance at *p* ≤ 0.05, 0.01, and 0.001, respectively. Relative content change (RCC) is calculated as [(WD − WW)/WW] × 100, using mean values. The mean DW/FW ratio was 17.56% for both treatments. Compound abbreviations are defined in Figure 5.

## Data Availability

The raw data supporting the conclusions of this article will be made available by the authors on request.

## Supplementary Materials

The following supporting information can be downloaded at: https://www.mdpi.com/article/10.3390/molecules31040712/s1, Figure S1: ^1^H-^1^H TOCSY of Sugarloaf chicory leaf extract in buffered D_2_O; Figure S2: ^1^H-^13^C HSQC of Sugarloaf chicory leaf extract in buffered D_2_O; Figure S3: ^1^H-^13^C HMBC of Sugarloaf chicory leaf extract in buffered D_2_O; Figure S4: ^1^H-^1^H TOCSY of Sugarloaf chicory root extract in buffered D_2_O; Figure S5: ^1^H-^13^C HSQC of Sugarloaf chicory root extract in buffered D_2_O; Figure S6: ^1^H-^13^C HMBC of Sugarloaf chicory root extract in buffered D_2_O; Figure S7: A fragment of DOSY spectrum of a Sugarloaf chicory root extract dissolved in buffered D_2_O. Table S1: Selected ^1^H NMR signals for integration and quantitative analysis. Reference [39] is cited in the supplementary materials.
