# Supplementary material for: Metabolic Profiling of Sugarloaf Chicory Roots: Structural Assignment of Sesquiterpene Lactone Conjugates and Response to Reduced Irrigation
Source: Molecules. 2026 Feb 19;31(4):712. doi: 10.3390/molecules31040712 (PMC12942909; doi:10.3390/molecules31040712)
Supplement: Supplementary file 1 [file molecules-31-00712-s001.zip › molecules-4141948-supplementary.pdf]

Supplementary Materials

Metabolic Profiling of Sugarloaf Chicory Roots: Structural Assignment of Sesquiterpene Lactone Conjugates and Response to Reduced Irrigation

Giuseppe Scioli [1,†], Lorenzo Pin [1,2,†], Giulio Testone [1], Anatoly Petrovich Sobolev [1,*] and Donato Giannino [1,*]

1) Institute for Biological Systems, Italian National Research Council, Monterotondo, 00015, Rome, Italy
2) Institute for Agricultural and Forestry Systems in the Mediterranean, Catania Unit, 95128,Catania, Italy

* Correspondence: anatoly.sobolev@cnr.it (A.P.S.); donato.giannino@cnr.it (D.G.)
† These authors contributed equally to this work.

Figure S1: ^{1}H-^{1}H TOCSY of sugarloaf chicory leaf extract in buffered D_2O

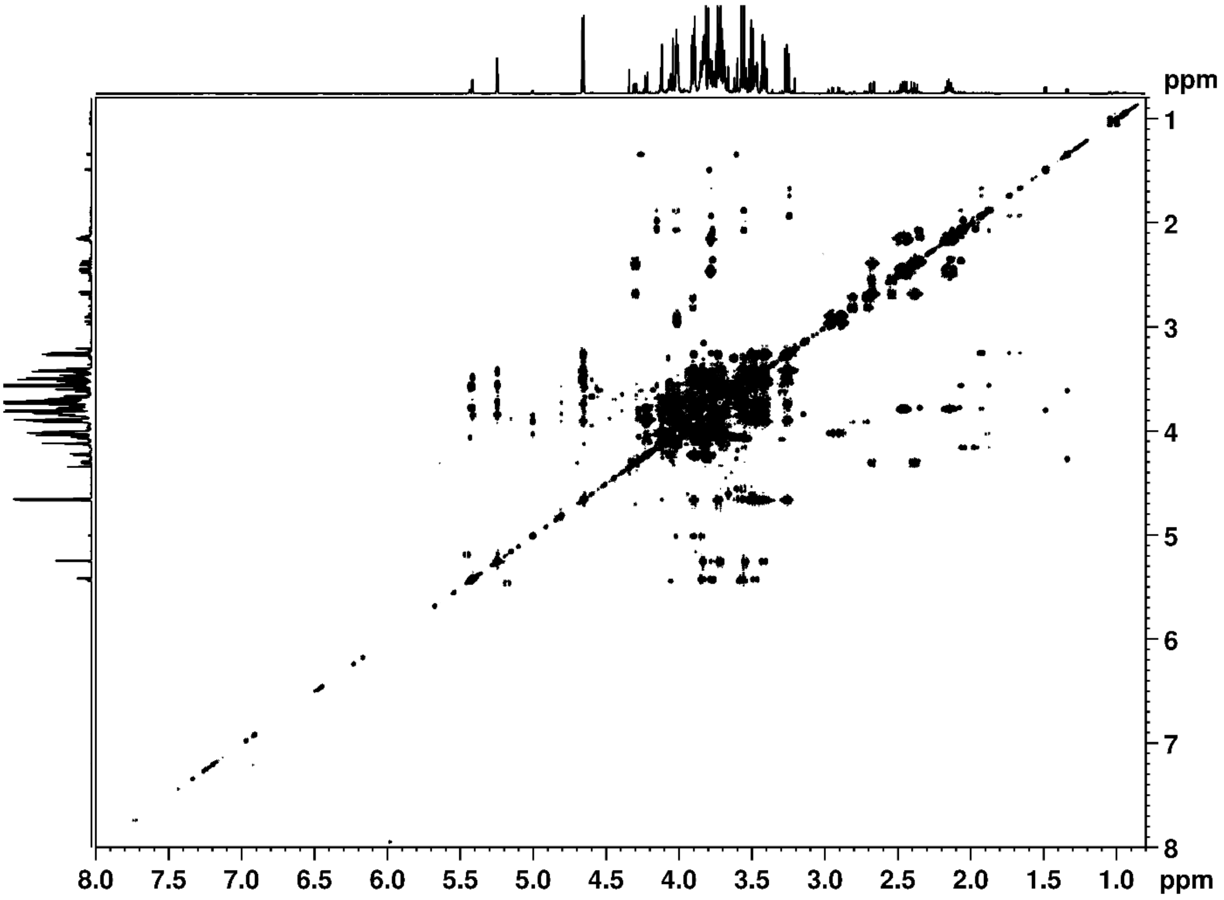

Figure S2: ^{1}H-^{13}C HSQC of sugarloaf chicory leaf extract in buffered D_2O

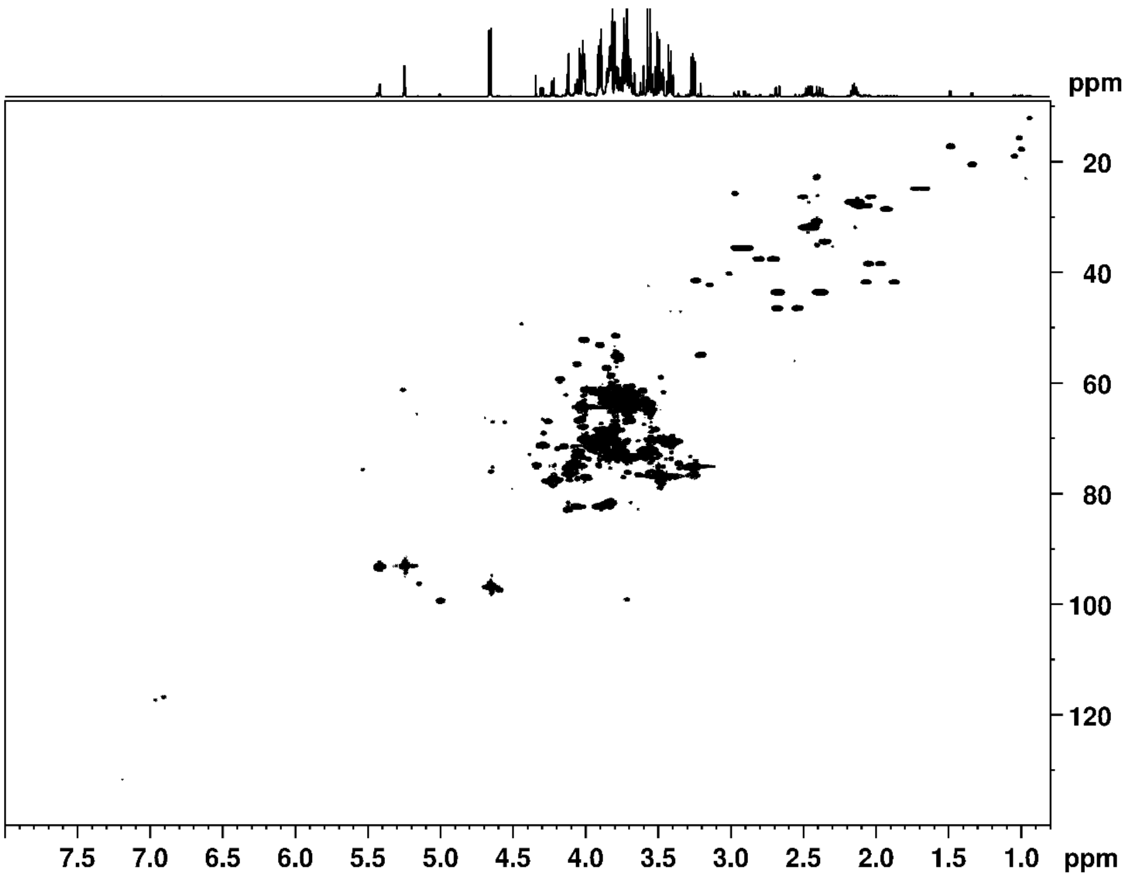

Figure S3: ^{1}H-^{13}C HMBC of sugarloaf chicory leaf extract in buffered D_2O

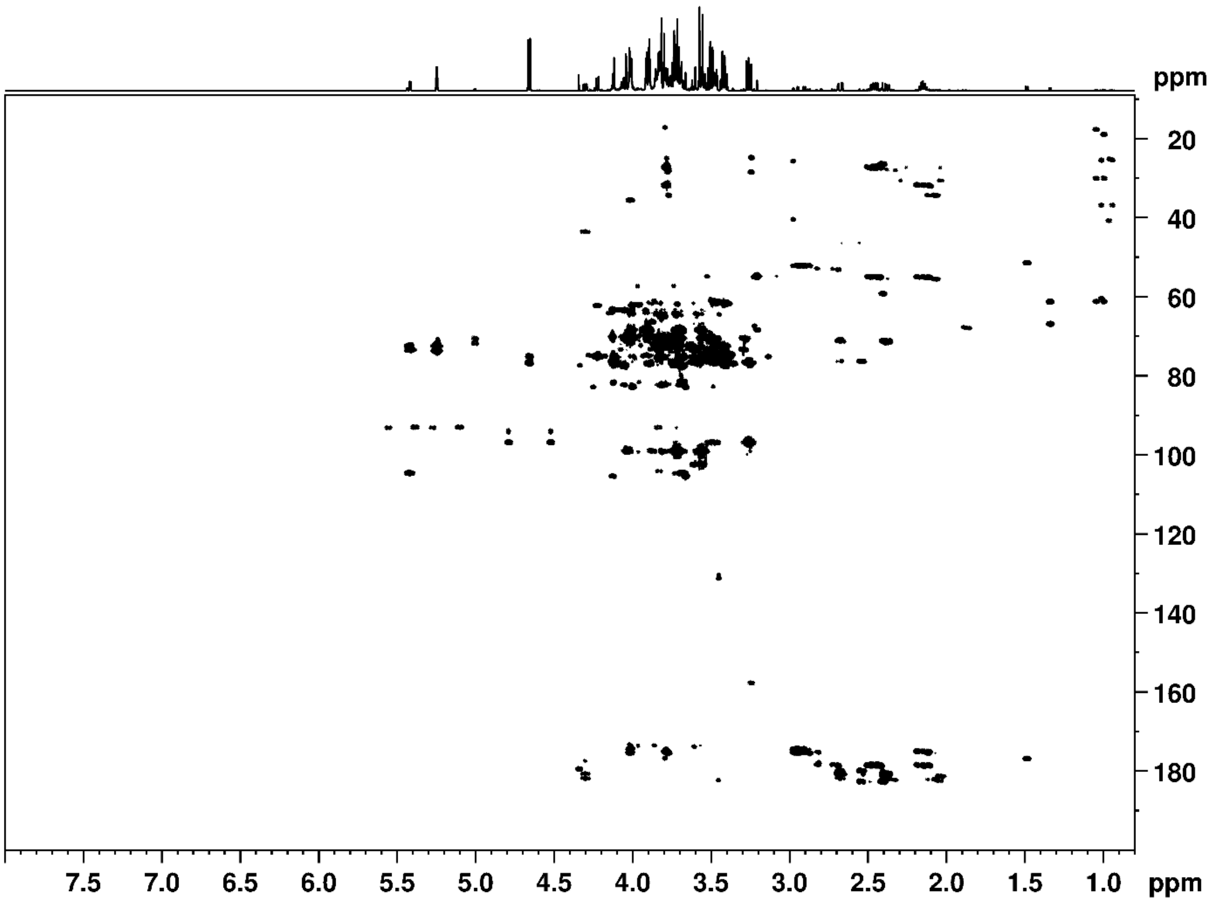

Figure S4: ^{1}H-^{1}H TOCSY of sugarloaf chicory root extract in buffered D_2O

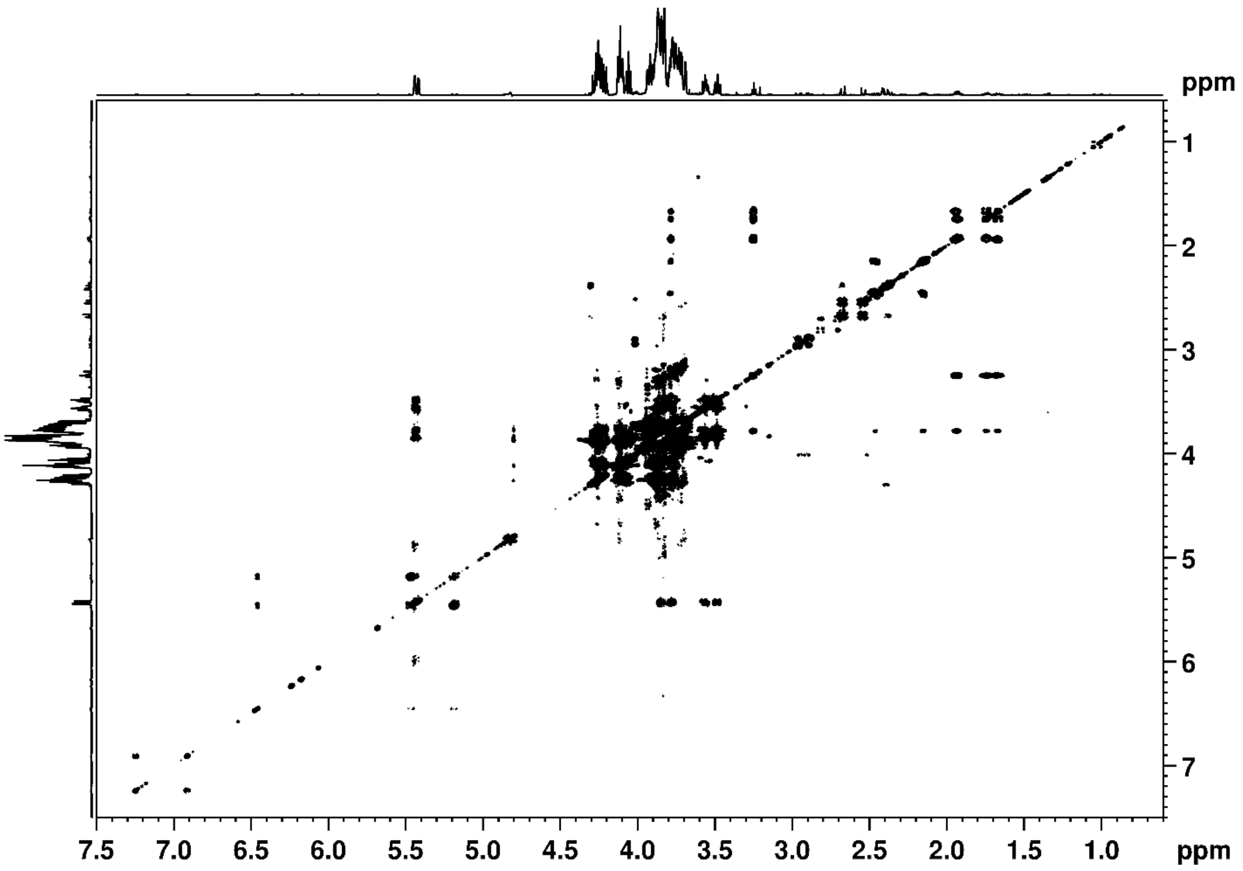

Figure S5: ^{1}H-^{13}C HSQC of sugarloaf chicory root extract in buffered D_2O

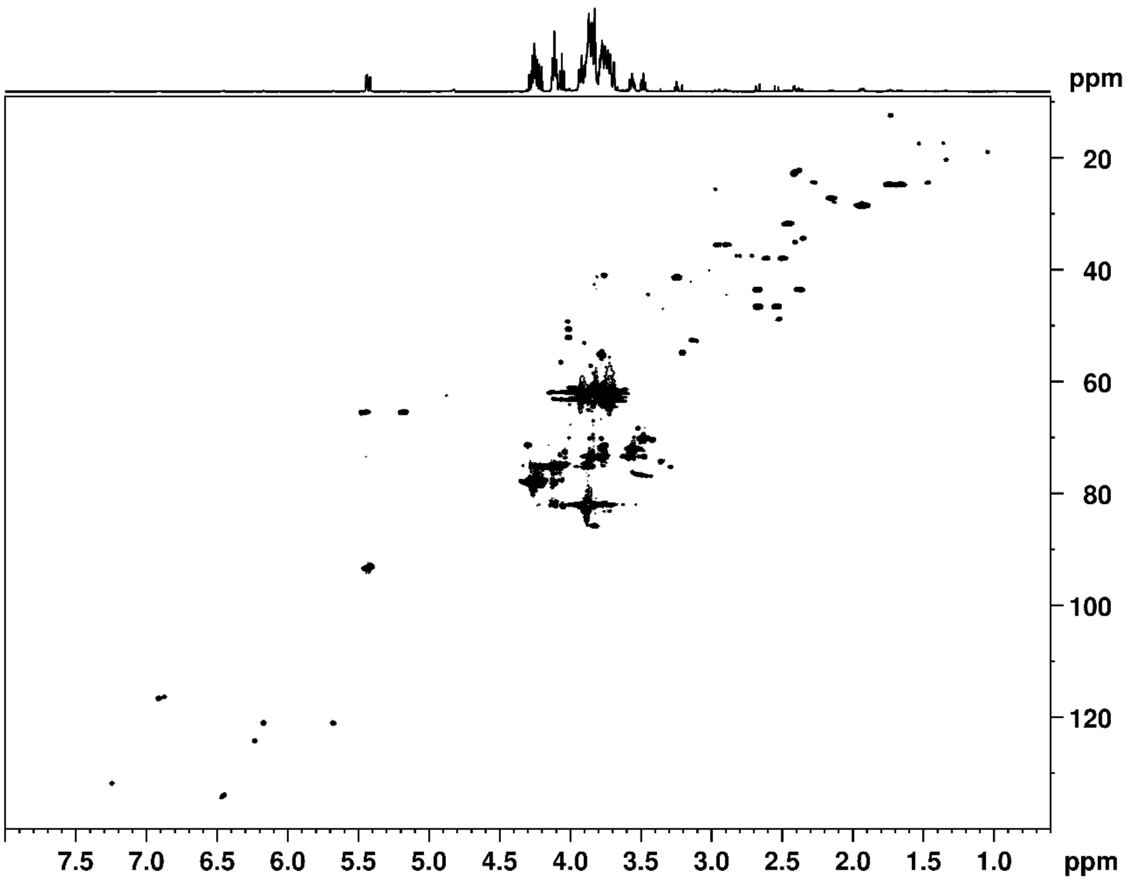

Figure S6: ^{1}H-^{13}C HMBC of sugarloaf chicory root extract in buffered D_2O

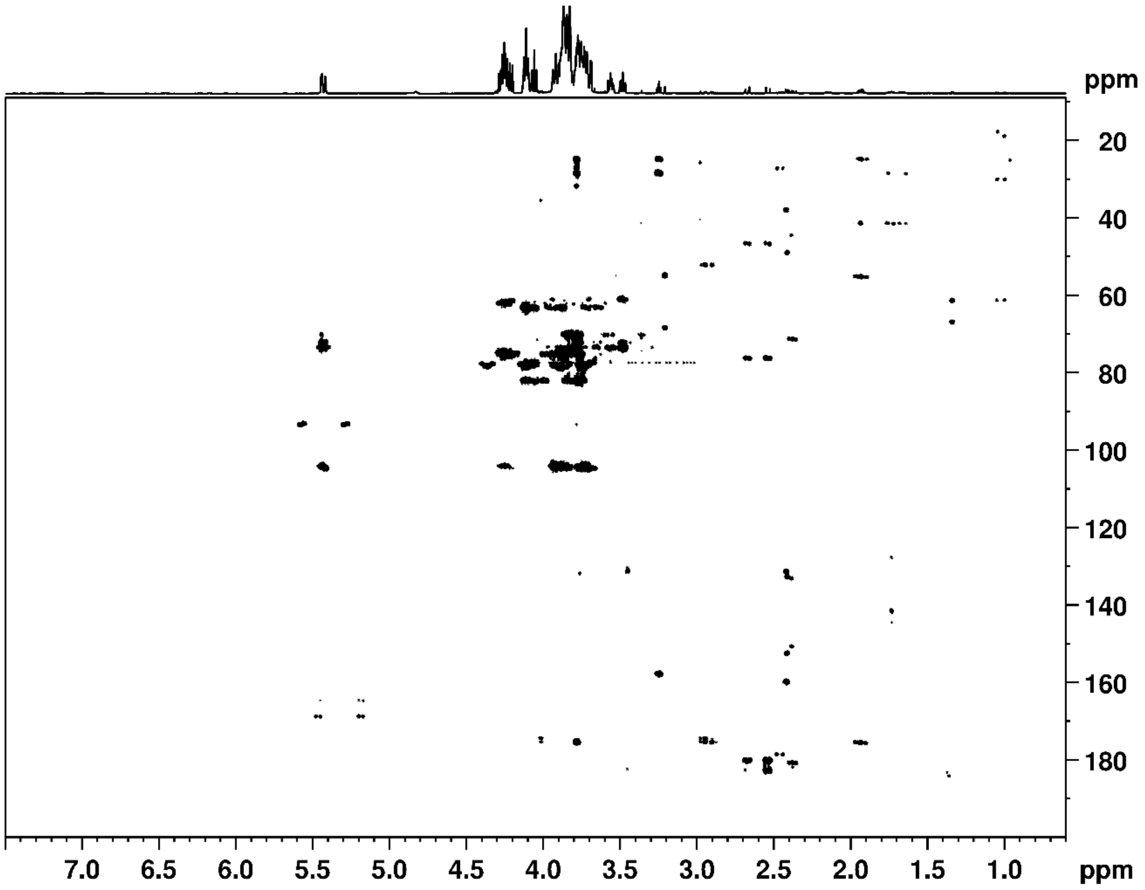

Figure S7: A fragment of DOSY spectrum of a sugarloaf chicory root extract dissolved in buffered D_2O

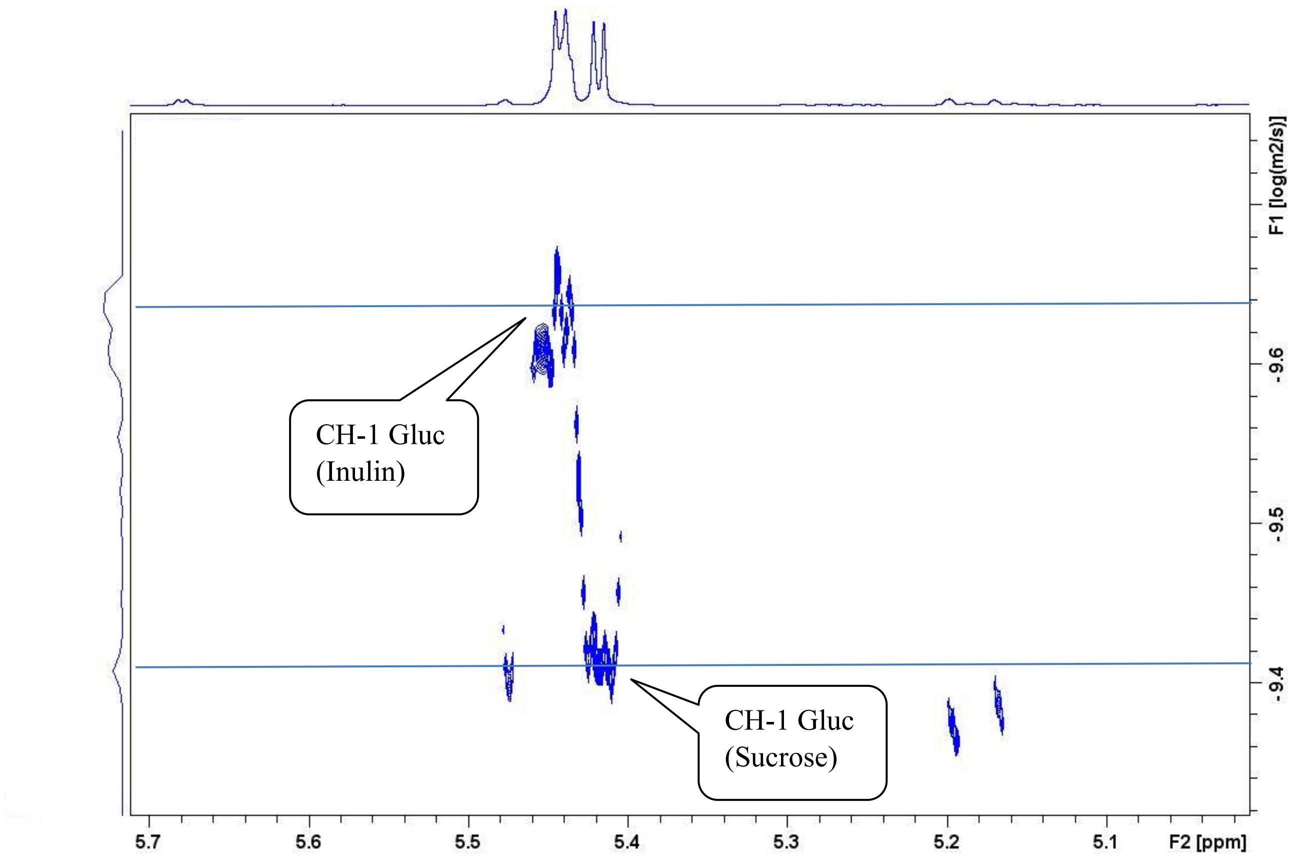

$D = 2.31 \cdot 10^{-10}$ m^2/s (Inulin); $D = 3.91 \cdot 10^{-10}$ m^2/s (Sucrose).

The diffusion coefficients (D) of uncharged polysaccharides are related to their molecular weights according to the empirical equation (Viel et al., 2003);

$D = 8.2 \cdot 10^{-9} \cdot M_w^{-0.49}$ (m^2/s), therefore

$M_w = (D/8.2 \cdot 10^{-9})^{-2.04}$

M_w (Inuline) = 1453,

$M_w(GF_n) = 180(1+n) - 18n = 180 + 162n$,

$n = (M_w-180)/162$

For $M_w = 1453$, $n = 7.9$

References

Viel, S.; Capitani, D.; Mannina, L.; Segre, A.L. Diffusion-ordered NMR spectroscopy: a versatile tool for the molecular weight determination of uncharged polysaccharides. *Biomacromolecules* **2003**, *4*, 1843-7. doi: 10.1021/bm0342638

Table S1. Selected ^{1}H NMR signals for integration and quantitative analysis.

Compound	Assignment	^{1}H (ppm)
Amino acids		
Alanine	$–CH_3$	1.48
Arginine	β–CH_2	1.67
Aspartic acid	β–CH	2.79
Asparagine	β'–CH	2.89
GABA	γ–CH_2	3.01
Glutamic acid	γ–CH	2.35
Glutamine	γ–CH	2.46
Histidine	–C=CH-4	8.12
Isoleucine	γ–CH_3	1.015
Leucine	$–CH_3$	0.961
Phenylalanine	–C=CH-3,5	7.43
Threonine	γ–CH_3	1.33
Tyrosine	–C=CH-2,6	7.12
Valine	$–CH_3$	1.04
Amino Alcohols		
Choline	$–N(CH_3)_3$	3.20
Phosphocholine	$–N(CH_3)_3$	3.22
Ethanolamine	$–CH_2(NH_2)$	3.14
Organic acids		
Citric acid	$–CH_2$	2.54
Formic acid	HCOOH	8.46
Fumaric acid	–C=CH	6.52
Lactic acid	$–CH_3$	1.33
Malic acid	–CH(OH)	4.29
Quinic acid	$–CH_2$-6	1.87
Succinic acid	$–CH_2–$	2.41
Tartaric acid	–CH(OH)	4.34
Sugars		
β-Glucose	–CH-1	4.66
α-Glucose	–CH-1	5.24
β-Galactose	–CH-1	4.59
α-Galactose	–CH-1	5.27
Fructose	–CH-4	4.12
Sucrose	–CH-1(Glu)	5.42
Raffinose	Glu-1	5.43
Inulin	Glu-1	5.44
Sugar Alcohols		
Myo-inositol	–CH-4	3.29
Chiro-inositol	–CH-3,4	3.59
Scyllo-inositol	–CH(OH)	3.35
Miscellaneous		
Trigonelline	–C=CH-4,6	8.84
Uridine	–C=C–H-6	7.94
